# Fracture Incidence and the Relevance of Dietary and Lifestyle Factors Differ in the United Kingdom and Hong Kong: An International Comparison of Longitudinal Cohort Study Data

**DOI:** 10.1007/s00223-021-00870-z

**Published:** 2021-06-03

**Authors:** Richard P. G. Hayhoe, Ruth Chan, Jane Skinner, Jason Leung, Amy Jennings, Kay-Tee Khaw, Jean Woo, Ailsa A. Welch

**Affiliations:** 1 Department of Epidemiology and Public Health, Norwich Medical School, Faculty of Medicine and Health Sciences, University of East Anglia, Norwich, NR4 7TJ UK; 2 School of Allied Health, Faculty of Health, Education, Medicine and Social Care, Anglia Ruskin University, Bishops Hall Lane, Chelmsford, CM1 1SQ UK; 3 Department of Medicine and Therapeutics, The Chinese University of Hong Kong, Shatin, New Territories, Hong Kong SAR China; 4 Jockey Club Centre for Osteoporosis Care and Control, The Chinese University of Hong Kong, Shatin, New Territories, Hong Kong SAR China; 5 Department of Public Health and Primary Care, Institute of Public Health, Strangeways Research Laboratory, University of Cambridge, Worts Causeway, Cambridge, CB1 8RN UK; 6 Jockey Club Institute of Ageing, The Chinese University of Hong Kong, Shatin, New Territories, Hong Kong SAR China

**Keywords:** Fractures, Osteoporosis, Nutrition, Epidemiology

## Abstract

Geographic variation in fracture risk may be due to divergent profiles of dietary, lifestyle, and other risk factors between populations. We investigated differences in fracture rates between two older-population cohorts: the European Prospective Investigation into Cancer and Nutrition (EPIC) Norfolk cohort (*n* = 7732) in the United Kingdom (UK), and the Mr and Ms Os cohort (*n* = 3956) in Hong Kong (HK). Data were collected by questionnaires, laboratory assessments, and hospital records. Incidence of hip, spine, and wrist fractures in the two cohorts was calculated and multivariable regression was used to explore variables important to fracture risk. Total hip, spine, and wrist fracture incidence was higher in the UK vs HK for women (13.70 vs 8.76 per 1000 person-years; *p* < 0.001), but not men (5.95 vs 5.37 per 1000 person-years; *p* = 0.337), and the proportions of different fractures also varied between cohorts (*p* < 0.001). Hip fracture was the most common UK fracture (accounting for 56.8% fractures in men and 52.6% in women), while wrist fracture was most common in HK (42.9% in men and 57.9% in women). The major contributor to total fracture risk in multivariable regression models of both cohorts and sexes, was age; with BMI also an important contributor to fracture risk HK men and UK women. The distribution of factors relevant to fracture risk, and the rates of different fractures, varied significantly between UK and HK cohorts. However, the importance of each factor in contributing to fracture risk was similar between the cohorts. The differences in fracture rates suggest targeted approaches may be required when developing interventions and public health recommendations to reduce the burden of osteoporosis in these two countries.

**Supplementary Information:**

The online version contains supplementary material available at 10.1007/s00223-021-00870-z.

## Introduction

Worldwide there are estimated to be nearly 9 million osteoporotic fractures annually, creating an enormous health care burden and representing a leading cause of disability [1]. The public health importance of the issue is made starkly apparent by the International Osteoporosis Foundation estimate that 1 in 3 women and 1 in 5 men over the age of 50 years will experience osteoporotic fractures [2]. However, osteoporotic fracture rates are known to vary significantly across the world [1]. Western populations have tended to present with higher incidence of fractures, but rapid increases in development and urbanisation in other countries, in particular Eastern Asia, have been associated with increasing incidence of fractures [3]. Genetic and ethnicity factors may be important in this variation. Indeed, differences in skeletal size and structure, bone microarchitecture, peak bone mineral density, and rate of bone loss during ageing, may all contribute to variation in fracture rates in different regions of the world [4]. This is particularly noticeable in comparisons between African Americans or New Zealand Maoris who have higher BMD and lower hip fracture rates than age-matched Caucasians [5].

Other environmental and modifiable factors such as lifelong diet and physical activity habits are also likely to play a role in determining fracture rates, and are more useful targets for potential intervention strategies. Dietary calcium and vitamin D have previously been the focus of nutritional interventions for bone health. These are particularly important during bone development [6], but their importance in older individuals has been questioned by a number of recent studies [7–9], and other nutrients, in particular micronutrients and antioxidants abundant in fruit and vegetables are gaining more attention as being potentially important [10, 11].

We have chosen to study differences in fracture rates and the associated demographic, dietary, and lifestyle factors involved in the United Kingdom (UK) and Hong Kong (HK). By investigating data from different countries with potentially different risk factors for osteoporotic fractures, we hope to further our understanding of the key influences that may help prevent or reduce fractures in older people. This comparative epidemiology approach will allow interpretation of the similarities and differences between the different population groups with different demographics and different incidences of disease. We know that the typical Chinese diet consists of a higher proportion of fruits and vegetables, and a lower intake of animal foods than a Western diet [12]. In addition, in comparison to the UK, previous research has demonstrated low intake of dietary calcium and dairy products in older individuals in HK [13], and likewise differences in vitamin D food intake and physical activity patterns [14], which may translate into differences in fracture incidence. The current study was thus designed to explore potential differences in the associations of a range of dietary and lifestyle factors with the risk of fractures, standardised for comparison, in those aged 65 years and over in a general United Kingdom population of older men and women, and a similar aged population in Hong Kong.

## Materials and Methods

The methods below describe the data collection and analysis techniques used for each cohort. Prior to commencing our statistical modelling, we ensured that all variables were coded as appropriately as possible to enable direct comparison between datasets. The EPIC-Norfolk dataset includes a large number of individuals below the age of 65 years, so these individuals were excluded from analyses to enable direct comparison with the HK dataset which contains only data of individuals 65 years or older.

### Participants

*United Kingdom*—The European Prospective Investigation into Cancer and Nutrition (EPIC) was established as a collaboration involving ten Western Europe countries. EPIC-Norfolk is one of the UK sub-cohorts, described in detail previously [15]. The EPIC-Norfolk sub-cohort has a wider remit than the overall diet and cancer study of EPIC and includes a focus on investigating modifiable lifestyle factors as determinants of chronic disease, disability and death in middle and later life. A general population sample was established through recruitment of individuals registered with a National Health Service general practitioner in Norfolk and resulted in 25,639 free-living men and women aged 39–79 years attending a baseline health-check between 1993 and 1997, and 15,786 participants aged 42–82 years attending a second health-check between 1998 and 2000. Our longitudinal analyses used data from the first health-check together with data of hospital recorded fractures for cohort participants (all cohort hip, spine, and wrist fracture cases up to 31^st^ March 2016; follow-up time was calculated as the time between an individual’s first health-check and this cut-off date, or death if earlier). Data for diet and fracture analyses were available for 3678 men and 4054 women aged 65 years and older.

*Hong Kong*—Subjects were participants of a prospective cohort study examining the risk factors for osteoporosis in Hong Kong [16]. A total of 2000 men and 2000 women aged 65 years and older of Chinese origin living in the community were recruited between 2001 and 2003 by placing recruitment notices in community centres for the older people and housing estates, using a stratified sample so that approximately 33% would be in each of these age groups: 65–69, 70–74, or 75 years and over. Participants were volunteers and were able to walk or take public transport to the study site. Compared with the general population of this age group, participants had higher educational level (9.8% vs. 3.8% with tertiary education), higher proportion of being married (70.7% vs. 59.9%), slightly lower proportion of living alone (10.8% vs. 11.3%) but similar duration of residence in Hong Kong (98.3% vs. 97.1% with duration at or over 15 years) [17]. This study followed the guidelines laid down in the Declaration of Helsinki, and was approved by the Clinical Research Ethics Committee of the University. Written informed consent was obtained from all participants. Those who had incomplete dietary data or extreme energy intake were excluded from the analysis (*n* = 44). The final sample of the present study included data from 3956 participants (1979 men and 1977 women).

### Anthropometric Measurements

*United Kingdom*—At each health-check height and body weight were recorded (to the nearest 1 mm and 0.2 kg, respectively) according to standard protocols, with participants wearing light clothing and no shoes [15].

*Hong Kong*—Similar standardised protocols were used to measured height to the nearest 1 mm and weight to the nearest 0.1 kg.

Body mass index (BMI) was calculated as body weight in kg / (height in m)^2^.

### Dietary Assessment

*United Kingdom*—All participants were asked to complete a self-administered food-frequency questionnaire (FFQ) [18]. The FFQ estimated habitual intake during last 12 months, with data collected on each food item, the size of each portion, and the number of times of consumption each day and each week. The intakes of specific nutrients were then computed with the use of an in-house programme, CAFE (Compositional Analyses from Frequency Estimates) [19].

*Hong Kong*—In a similar way, dietary intake was assessed at baseline using a validated FFQ [20]. Mean nutrient quantitation per day and daily consumption of various food groups were calculated using food composition tables derived from McCance and Widdowson [21] and the Chinese Medical Sciences Institute [22].

### Assessment of Fracture Risk

*United Kingdom*—Fracture incidence data were ascertained using record linkage with the East Norfolk COmmission REcord (ENCORE) of hospital attendances by Norfolk residents [23]. Incidence of all fractures in the cohort, up to 31^st^ March 2016, was thus determined by retrieving data using each participant’s NHS number and searching for events logged using International Classification of Diseases 9 and 10 diagnostic codes for osteoporotic hip, spine, or wrist fractures. Pre-2009 data were derived from a Hospital Episode Statistic (HES) database maintained locally by the Norfolk Primary Care Trust (PCT); post-2009 data were acquired from national HES databases held by NHS Digital. Total risk of hip, spine, or wrist fracture was calculated as the risk of the first occurrence of one of these fractures; this does not consider multiple fractures and therefore the sum of the specific-site fracture incidences does not sum to the total.

*Hong Kong*—Data on incident fractures of hip, spine, and wrist were obtained by carrying out a search of the Hospital Authority electronic database, which covers over 95% of all hospital admissions in Hong Kong. Incident fractures, up to 31^st^ October 2013, were documented from hospital databases and all patient records were reviewed by clinicians.

Follow-up time was calculated as the time between an individual’s first health-check and the fracture record search, or death if earlier.

### Other Covariates

For both cohorts, health and lifestyle questionnaires were used to collect baseline information on age, gender, education level, smoking habit, alcohol use, use of calcium supplements, use of hormonal replacement therapy (HRT) (for women only), and family history of osteoporosis. Physical activity in the Hong Kong cohort was assessed by the Physical Activity Scale for the Elderly (PASE) [24]. A composite PASE score of all the items was calculated, a higher score reflecting higher physical activity level. These data were transformed into categorical data to match the United Kingdom cohort, placing participants into *inactive*, *moderately inactive*, *moderately active*, and *active* categories by a method validated against heart-rate monitoring data [25]. Fasting peripheral blood venous samples were collected and serum isolated for assay of 25-OH-vitamin D (25-OHD). Serum samples were frozen until being thawed immediately prior to assay of 25-OHD concentration using a competitive radioimmunoassay (DiaSorin, Stillwater, USA) for Hong Kong samples, or an ultraperformance liquid chromatography interfaced by atmospheric pressure chemical ionisation to mass spectrometry method (VITAS, Oslo, Norway) for United Kingdom samples. Both methods assessed 25-OH-vitamin D2 and 25-OH-vitamin D3; the total of D2 and D3 isoforms has been used in our analyses.

### Statistical Analyses

The High-Performance Computing Cluster supported by the Research and Specialist Computing Support service at the University of East Anglia was used for statistical data analysis with STATA (v.15; Stata Corp., Texas) and R (v.4.0.3) software. Sex stratification has been used in all our analyses. Any *p* values < 0.05 were therefore considered to be statistically significant in individual analyses. Fracture incidence rates per 1000 person-years were calculated for total, hip, spine, or wrist fractures in both cohorts by the following formula: number of fractures (first occurrence only) /number of person-years × 1000. Two-sided exact significance testing was carried out to determine differences between rates in the two cohorts. Cox regression was used to investigate individual associations between incidence of fractures (total, hip, spine, or wrist) and age, BMI, physical activity, smoking status, family history of osteoporosis, education, dietary calcium intake, dietary vitamin D intake, vegetable consumption, fruit consumption, alcohol consumption, use of calcium supplements and HRT status in women [26, 27]. Total risk of hip, spine, or wrist fracture was calculated as the risk of the first occurrence of one of these fractures; this does not consider multiple fractures and therefore the sum of the specific-site fracture incidences does not sum to the total. Follow-up time was calculated as the time between an individual’s first health-check and this cut-off date, or death if earlier.

**Fig. 1:**
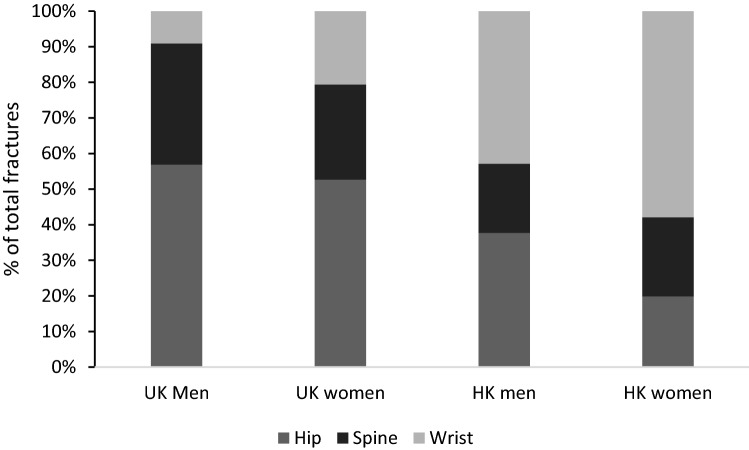
Proportion of hip, spine, and wrist fractures in men and women of the UK and HK cohorts

**Fig. 2:**
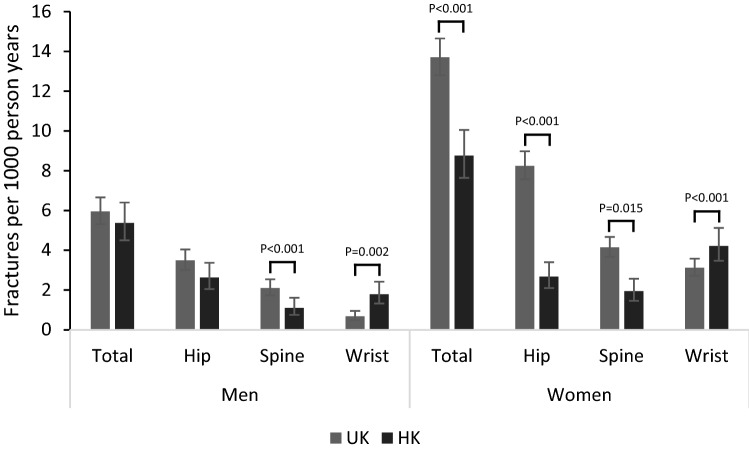
Incident fracture risks in men and women of the UK and HK cohorts. *p* values for two-sided exact significance testing of rates in the two cohorts. Data used to generate the standardised fracture rates are available in Supplementary Table 1.

A single multivariable model was used, fitting all of the risk factors, and calculating their contribution to the model in terms of Heller’s measure of R^2^ [28]. Since hazard ratios are dependent on scale, using the R^2^ statistic provides a useful additional measure that indicates the proportion of variability in the outcome explained by the model variables. An omnibus *p* value for fitting each variable as a whole (for example, all of the BMI group dummy variables) was calculated, based on testing the change in deviance on the appropriate degrees of freedom. The variables included were decided according to previously established risk factors. Except for the direct comparison between cohorts of differences in the demographic, biological, and lifestyle variables in Figs. 1 and 2, we analysed the two cohorts separately. This was primarily in order to be able to determine which variables were important at explaining fracture risk for each cohort, irrespective of whether there might be similar proportions for a particular variable between the two cohorts and thus no contribution to a difference in incidence. We also carried out sub-analyses restricting the cohort samples to specific age ranges to allow direct comparison, and additionally compared older vs younger individuals in the UK and HK cohorts, respectively, to explore the relationship of age in determining differences in the proportions of hip, spine, and wrist fractures between cohorts. We tested whether the assumption of proportional hazards was valid using scaled Schoenfeld residuals (using estat phtest in Stata).

## Results

Selected characteristics of the United Kingdom and Hong Kong cohort populations are summarised in Table 1 for men, and Table 2 for women. Data were available for 3678 men and 4054 women in the United Kingdom, and 1979 men and 1977 women in Hong Kong, aged 65 years or older. Significant differences between the UK and HK cohorts were seen for all variables tested for both men and women, except for fruit consumption in women (*p* = 0.062, *n* = 6031). Compared to the UK cohort, men in the HK cohort had lower BMI; were less physically active; were more likely to have never smoked; had lower prevalence of family history of osteoporosis; were more likely to have no secondary education; had lower dietary calcium, vitamin D, and alcohol intakes, but higher fruit and vegetable intake; more were taking calcium supplementation; and more had adequate serum 25-OHD. Differences between the cohorts for women were similar to the difference seen for men except that in the HK cohort there were proportionally even fewer women who were current or past smokers, family history of osteoporosis was less different, and HRT use was much lower (Tables 3 and 4).

**Table 1:** Baseline characteristics of men in the UK (*n* = 3678) and HK (*n* = 1979) cohorts

Parameter	UK (*n* = 3678)				HK (*n* = 1979)				
	Mean	SD	*n*	%	Mean	SD	*n*	%	*p* value
Age (years)	70.4	3.3			72.4	5.0			< 0.001
*Age group* (years), *n* (%)									< 0.001
< 70y			1812	49.3			660	33.4	
≥ 70y and < 75y			1505	40.9			699	35.3	
≥ 75y and < 80y			361	9.8			439	22.2	
> 80y			–	–			181	9.2	
BMI (kg/m^2^)	26.7	3.27			23.4	3.1			< 0.001
*BMI categories* (kg/m^2^), *n* (%)									< 0.001
< 18.5			11	0.3			113	5.7	
≥ 18.5 and < 25 UK; ≥ 18.5 and < 23 HK			1133	30.8			1274	64.4	
≥ 25 and < 30 UK; ≥ 23 and < 25 HK			2011	54.7			556	28.1	
≥ 30 UK; ≥ 25 HK			523	14.2			36	1.8	
*Physical activity level*, *n* (%)									< 0.001
Active			481	13.1			202	10.2	
Moderately active			647	17.6			162	8.2	
Moderately inactive			932	25.3			592	29.9	
Inactive			1618	44.0			1023	51.7	
*Smoking status*, *n* (%)									< 0.001
Never			888	24.1			715	36.1	
Current or former			2790	75.9			1264	63.9	
*Family Hx of osteoporosis*, *n* (%)									< 0.001
No			3612	98.2			1868	94.4	
Yes			66	1.8			111	5.6	
*Education*, *n* (%)									< 0.001
None or pre-secondary education only			1556	42.3			1191	60.2	
Secondary, college, or further education			1745	47.4			518	26.2	
Higher education/university			377	10.3			270	13.6	
Dietary calcium intake (mg/1000 kcal)	486.0	113.2			299.6	116.5			< 0.001
Dietary calcium intake ≥ 700 mg/d, *n* (%)			3250	88.4			670	33.9	< 0.001
Dietary vitamin D intake (ug/1000 kcal)	1.70	0.79			0.17	0.26			< 0.001
Vegetable consumption (g/1000 kcal/d)	114.5	58.6			118.3	74.9			0.037
Fruit consumption (g/1000 kcal/d)	108.3	79.1			132.5	88.6			< 0.001
Ethanol consumption (g/d)	1.33	1.82			0.2	0.86			< 0.001
*Ethanol consumption*, *n* (%)									< 0.001
0 units			674	18.3			1514	76.5	
> 0 and ≤ 2 units UK; > 0 HK			2265	61.6			465	23.5	
≥ 2 units UK			739	20.1					
*Use of calcium supplement*, *n* (%)									< 0.001
No			3625	98.6			1788	90.4	
Yes			53	1.4			191	9.7	
*Serum vitamin D* (nmol/L)^a^	55.6	21.2			63.3	15.6			< 0.001
≥ 50 nmol/L, *n*(%)			817	56.9			1108	79.7	< 0.001

^a^Serum vitamin D data were available for 1435 UK and 1396 HK men. *P* values for differences between UK and HK cohorts according to t-test for continuous or Chi-square for categorical variables

**Table 2:** Baseline characteristics of women in the UK (*n* = 4054) and HK (*n* = 1977) cohorts

Parameter	UK (*n* = 4054)				HK (*n* = 1977)				
	Mean	SD	*n*	%	Mean	SD	*n*	%	*p* value
Age (years)	70.3	3.3			72.5	5.3			< 0.001
*Age group* (years), *n* (%)									< 0.001
< 70y			2027	50.0			665	33.6	
≥ 70y and < 75y			1664	41.1			659	33.3	
≥ 75y and < 80y			363	9.0			443	22.4	
> 80y			–	–			210	10.6	
BMI (kg/m^2^)	26.6	4.1			23.9	3.4			< 0.001
BMI categories (kg/m^2^), *n* (%)									< 0.001
≥ 18.5 and < 25 UK; ≥ 18.5 and < 23 HK			31	0.8			95	4.8	
≥ 25 and < 30 UK; ≥ 23 and < 25 HK			1478	36.5			1177	59.5	
≥ 30 UK; ≥ 25 HK			1817	44.8			622	31.5	
≥ 18.5 and < 25 UK; ≥ 18.5 and < 23 HK			728	18.0			83	4.2	
*Physical activity level*, *n* (%)									< 0.001
Active			312	7.7			80	4.1	
Moderately active			572	14.1			71	3.6	
Moderately inactive			1246	30.7			416	21.0	
Inactive			1924	47.5			1410	71.3	
*Smoking status*, *n* (%)									< 0.001
Never			358	53.8			1789	90.5	
Current or former			3696	46.2			188	9.5	
*Family Hx of osteoporosis*, *n* (%)									0.013
No			3911	96.5			1881	95.1	
Yes			143	3.5			96	4.9	
*HRT use*, *n* (%)									< 0.001
Never			3500	86.3			1914	96.8	
Past			312	7.7			53	2.7	
Current			242	6.0			10	0.5	
*Education*, *n* (%)									< 0.001
None or pre-secondary education only			2290	56.5			1635	82.7	
Secondary, college, or further education			1512	37.3			223	11.3	
Higher education/university			252	6.2			119	6.0	
Dietary calcium intake (mg/1000 kcal)	519.0	121.3			361.3	136.4			< 0.001
Dietary calcium intake ≥ 700 mg/d, *n* (%)			3483	85.9			526	26.6	< 0.001
Dietary vitamin D intake (ug/1000 kcal)	1.79	0.83			0.19	0.21			< 0.001
Vegetable consumption (g/1000 kcal/d)	140.0	74.0			151.6	86.4			< 0.001
Fruit consumption (g/1000 kcal/d)	150.3	99.4			155.2	91.5			0.062
Ethanol consumption (g/d)	0.61	0.99			0.01	0.09			< 0.001
*Ethanol consumption*, *n* (%)									< 0.001
0 units			1309	32.3			1926	97.4	
> 0 and ≤ 2 units UK; > 0 HK			2450	60.4			51	2.6	
≥ 2 units UK			295	7.3					
*Use of calcium supplement*, *n* (%)									< 0.001
No			3885	95.8			1623	82.1	
Yes			169	4.2			354	17.9	
*Serum vitamin D* (nmol/L)^a^	50.5	21.2			57.8	14.6			< 0.001
≥ 50 nmol/L, *n* (%)			683	46.2			920	66.2	< 0.001

^a^Serum vitamin D data were available for 1477 UK and 1389 HK women. *p* values for differences between UK and HK cohorts according to t-test for continuous or Chi-square for categorical variables

**Table 3:** Multivariate Cox regression results in men linking contributory factors to the total risk of hip, spine and wrist fractures in the UK and HK cohorts

Characteristic	UK men					HK men				
	HR	95% CI	*p* value	Omnibus *p*	*R*^2^	HR	95% CI	*p* value	Omnibus *p*	*R*^2^
*Age group*				< 0.001	0.1126				< 0.001	0.1127
< 70	1.00	–				1.00	–			
70 to < 75	2.09	1.64, 2.67	< 0.001			1.68	1.02, 2.76	0.042		
75 to < 80	2.57	1.73, 3.83	< 0.001			2.61	1.54, 4.40	< 0.001		
≥ 80 HK						3.29	1.78, 6.08	< 0.001		
*BMI categories* (kg/m^2^)				0.437	0.0075				0.014	0.0511
< 18.5	1.95	0.27, 14.1	0.507			2.07	1.16, 3.71	0.014		
18.5 to < 25 UK; 18.5 to < 23 HK	1.00	–				1.00	–			
25 to < 30 UK; 23 to < 25 HK	0.82	0.64, 1.06	0.132			0.67	0.41, 1.10	0.114		
≥ 30 UK; ≥ 25 HK	0.90	0.62, 1.30	0.564			0.84	0.54, 1.31	0.447		
*Physical activity level*				0.874	0.0016				0.550	0.0071
Inactive	1.00	–				1.00	–			
Moderately inactive	1.02	0.77, 1.36	0.869			1.10	0.74, 1.64	0.626		
Moderately active UK; Active/moderately active HK	0.90	0.65, 1.24	0.510			0.81	0.47, 1.40	0.444		
Active UK	1.03	0.73, 1.45	0.880							
*Smoking status*				0.230	0.0036				0.309	0.0060
Never smoked	1.00	–				1.00	–			
Current or former smoker	1.18	0.90, 1.55	0.236			1.22	0.83, 1.81	0.314		
*Family Hx of osteoporosis*				0.129	0.0050				0.965	0.0000
No	1.00	–				1.00	–			
Yes	1.75	0.90, 3.43	0.100			1.02	0.45, 2.32	0.964		
*Education*				0.681	0.0018				0.283	0.0148
None/pre-secondary	1.00	–				1.00	–			
Secondary/further education	1.02	0.80, 1.30	0.889			0.71	0.45, 1.13	0.148		
Higher education	0.85	0.57, 1.29	0.454			1.05	0.62, 1.80	0.848		
*Dietary Ca meeting RNI*				0.303	0.0028				0.515	0.0024
No	1.00	–				1.00	–			
Yes	0.83	0.59, 1.18	0.293			1.14	0.78, 1.66	0.513		
Dietary vitamin D intake (ug/1000 kcal)	1.01	0.87, 1.17	0.904	0.904	0.0000	1.30	0.75, 2.24	0.351	0.389	0.0034
Vegetable consumption (g/100 kcal/d)	1.00	0.98, 1.02	0.921	0.921	0.0000	1.01	0.99, 1.04	0.166	0.193	0.0090
Fruit consumption (g/100 kcal/d)	1.01	0.99, 1.02	0.396	0.402	0.0022	0.98	0.96, 1.01	0.192	0.171	0.0116
*Ethanol consumption* (units/d)				0.362	0.0052				0.847	0.0002
None	1.00	–				1.00	–			
> 0 to < 2 UK; > 0 HK	0.85	0.63, 1.16	0.306			1.04	0.68, 1.61	0.846		
≥ 2 UK	1.02	0.70, 1.48	0.919							
*Use of Ca supplement*				0.932	0.0000				0.995	0.0000
No	1.00	–				1.00	–			
Yes	1.04	0.43, 2.54	0.932			1.00	0.54, 1.83	0.995		

*HR* Hazard Ratio, *CI* Confidence Interval

**Table 4:** Multivariate Cox regression results in women linking contributory factors to the total risk of hip, spine and wrist fractures in the UK and HK cohorts

Characteristic	UK women					HK women				
	HR	95% CI	*p* value	Omnibus *p*	*R*^2^	HR^*1*^	95% CI^*1*^	*p* value	Omnibus *p*	*R*^2^
*Age group*				< 0.001	0.0732				< 0.001	0.0760
< 70	1.00	–				1.00	–			
70 to < 75	1.78	1.54, 2.06	< 0.001			1.69	1.15, 2.48	0.007		
75 to < 80	2.18	1.71, 2.77	< 0.001			2.06	1.38, 3.08	< 0.001		
≥ 80 HK						2.47	1.53, 3.97	< 0.001		
*BMI categories* (kg/m^2^)				< 0.001	0.0249				0.613	0.0060
< 18.5	2.12	1.12, 3.99	0.020			1.48	0.82, 2.64	0.190		
18.5 to < 25 UK; 18.5 to < 23 HK	1.00	–				1.00	–			
25 to < 30 UK; 23 to < 25 HK	0.77	0.66, 0.89	< 0.001			1.08	0.75, 1.56	0.674		
≥ 30 UK; ≥ 25 HK	0.67	0.54, 0.83	< 0.001			1.14	0.81, 1.59	0.450		
*Physical activity level*				0.218	0.0043				0.596	0.0040
Inactive	1.00	–				1.00	–			
Moderately inactive	0.86	0.74, 1.01	0.070			1.16	0.83, 1.62	0.389		
Moderately active UK; Active/moderately active HK	0.87	0.71, 1.07	0.186			0.88	0.49, 1.60	0.678		
Active UK	0.84	0.64, 1.10	0.205							
*Smoking status*				0.338	0.0009				0.502	0.0015
Never smoked	1.00	–				1.00	–			
Current or former smoker	1.07	0.93, 1.23	0.337			1.16	0.75, 1.80	0.495		
*Family Hx of osteoporosis*				0.667	0.0002				0.236	0.0054
No	1.00	–				1.00	–			
Yes	1.08	0.75, 1.57	0.663			1.46	0.81, 2.64	0.212		
*Education*				0.822	0.0004				0.420	0.0070
None/pre-secondary	1.00	–				1.00	–			
Secondary/further education	0.99	0.86, 1.15	0.906			0.73	0.44, 1.21	0.225		
Higher education	1.09	0.82, 1.44	0.563			1.07	0.59, 1.96	0.822		
*Dietary Ca meeting RNI*				0.583	0.0003				0.475	0.0020
No	1.00	–				1.00	–			
Yes	0.95	0.78, 1.15	0.581			0.89	0.64, 1.24	0.479		
Dietary vitamin D intake (ug/1000 kcal)	1.03	0.95, 1.12	0.434	0.437	0.0005	1.42	0.78, 2.57	0.248	0.263	0.0044
Vegetable consumption (g/100 kcal/d)	1.00	0.99, 1.01	0.802	0.803	0.0001	1.01	0.99, 1.02	0.373	0.391	0.0030
Fruit consumption (g/100 kcal/d)	1.00	0.99, 1.01	0.721	0.722	0.0001	1.00	0.98, 1.01	0.914	0.914	0.0000
*Ethanol consumption *(units/d)				0.009	0.0097				0.029	0.0308
None	1.00	–				1.00	–			
> 0 to < 2 UK; > 0 HK	0.87	0.75, 1.01	0.069			0.20	0.03, 1.41	0.105		
≥ 2 UK	0.63	0.45, 0.87	0.005							
*Use of Ca supplement*				0.725	0.0001				0.497	0.0017
No	1.00	–				1.00	–			
Yes	1.06	0.76, 1.49	0.723			1.13	0.80, 1.61	0.492		
*HRT use*				0.242	0.0013				0.530	0.0020
Never	1.00	–				1.00	–			
Past/current	1.13	0.92, 1.39	0.248			1.36	0.50, 3.73	0.549		

*HR* Hazard Ratio, *CI* Confidence Interval

### Proportions of Hip, Wrist, and Spine Fractures

The proportion of hip, spine, and wrist fractures were significantly different between the two cohorts, for both men (*p* < 0.001, *n* = 362) and women (*p* < 0.001, *n* = 1017) (see Fig. 1). In the UK cohort, hip fractures accounted for the largest proportion of fractures (56.8% in men and 52.6% in women); and wrist fractures made up the smallest proportion, particularly in men (9.1% in men and 20.7% in women). By contrast, in the HK cohort wrist fractures accounted for the largest proportion (42.9% in men and 57.9% in women); hip fractures made up 37.7% in men and 19.8% in women; and spine fractures accounted for 19.5% in men and 22.2% in women.

### Fracture Incidence Rates

Participants were followed-up for a mean ± SD of 15.12 ± 5.95 years in the UK cohort, and 9.94 ± 2.31 years in the HK cohort. Calculation of standardised fracture incidence rates for the two cohorts highlighted a number of significant differences for both men and women (see Fig. 2 and Supplementary Table 1). Total fracture rate was significantly higher in the UK vs HK for women (13.70 vs 8.76 per 1000 person-years; *p* < 0.001), but not men (5.95 vs 5.37 per 1000 person-years; *p* = 0.337). Similarly, hip fracture rates were significantly higher in the UK vs HK for women (8.24 vs 2.67 per 1000 person-years; *p* < 0.001), but not men (3.49 vs 2.63 per 1000 person-years; *p* = 0.053). Spinal fracture rates were higher in the UK vs HK for both women (4.14 vs 1.94 per 1000 person-years; *p* = 0.015) and men (2.10 vs 1.10 per 1000 person-years; *p* < 0.001). By contrast, wrist fracture rates were lower in the UK vs HK for both women (3.12 vs 4.22 per 1000 person-years; *p* < 0.001) and men (0.68 vs 1.79 per 1000 person-years; *p* < 0.001).

In a sub-analysis excluding individuals over the age of 75 years (UK: 3317 men and 3691 women; HK: 1480 men and 1421 women) (see Supplementary Table 2), all trends remained the same as in the main analyses, but total and hip fracture rates in men were additionally significantly different between the UK and HK cohorts. Thus, total fracture rates in the UK vs HK were higher for both women (13.28 vs 6.92 per 1000 person-years; *p* < 0.001) and men (5.77 vs 4.02 per 1000 person-years; *p* = 0.005). Hip fracture rates were significantly higher in the UK vs HK for women (8.00 vs 1.36 per 1000 person-years; *p* < 0.001) and men (3.46 vs 1.55 per 1000 person-years; *p* < 0.001). Spinal fracture rates were higher in the UK vs HK for both women (4.00 vs 1.52 per 1000 person-years; *p* < 0.001) and men (2.07 vs 0.80 per 1000 person-years; *p* < 0.001). Wrist fracture rates were lower in the UK vs HK for both women (3.09 vs 4.08 per 1000 person-years; *p* < 0.048) and men (0.55 vs 1.77 per 1000 person-years; *p* < 0.001).

### Factors Associated with Fracture Risk

**Fig. 3:**
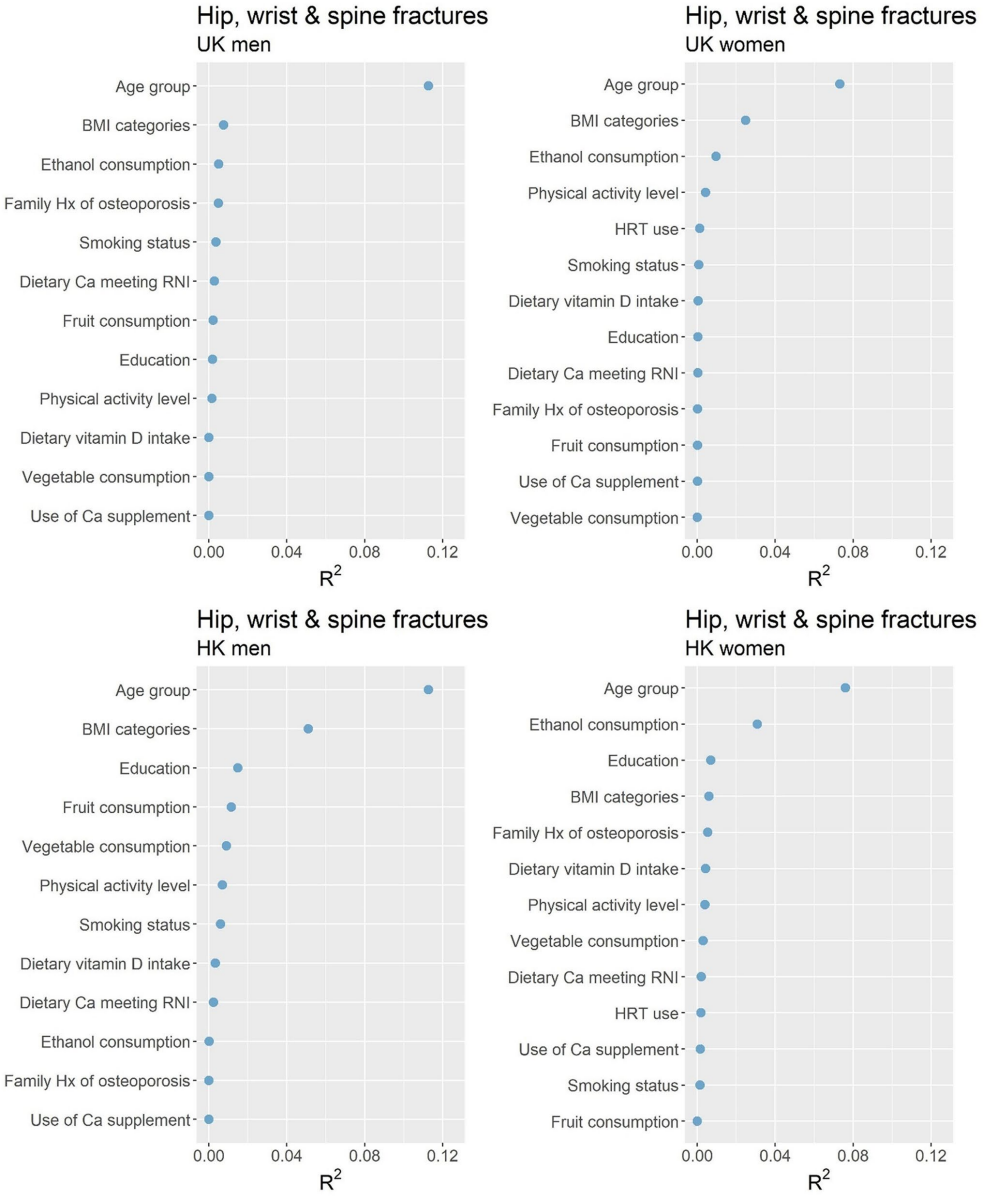
Contributions of individual factors to Cox regression model of risk of hip, spine and wrist fractures in the UK and HK cohorts, stratified by sex

A limited number of the variables chosen a priori to be included in our analyses proved to be significantly associated with fracture risk in either the UK or HK cohorts. To interpret the contributions of these significant factors, we used the R^2^ statistic and have presented this graphically in Fig. 3 (total fracture) and Supplementary Figs. 1, 2, and 3 (hip, spine, and wrist fractures, respectively); the total R^2^ gives an indication of how much the model explains the variance in fracture risk. For total fracture risk, age was a significant adverse factor in both the cohorts for both sexes (all *p* < 0.001), with older age groups associated with higher hazard ratios compared to the reference group of individuals under 70 years old (see Tables 3 and 4). Age was the major contributor to R^2^ in the regression model, accounting for 0.1126 for men and 0.0732 for women in the UK cohort, and 0.1127 and 0.0760 respectively in the HK cohort. BMI was associated with total fracture risk for men in the HK cohort (*p* = 0.014), contributing 0.0511 to the R^2^; being underweight (BMI < 18.5) was particularly detrimental to fracture risk with a hazard ratio of 2.07 (*p* = 0.014) compared to the normal weight reference group. In the UK cohort, BMI was also significant factor for fracture risk in women (*p* < 0.001) and contributed 0.0249 to the R^2^; being underweight had a hazard ratio of 2.12 (*p* = 0.020), while overweight and obese had hazard ratios of 0.77 (*p* < 0.001) and 0.67 (*p* < 0.001), respectively. The only other factor significantly associated with total fracture risk in the multivariable model was alcohol consumption in UK women (*p* = 0.009), where those consuming 2 or more units per day had a hazard ratio of 0.63 (*p* = 0.005) compared to those consuming none; this contributed 0.0097 to the R^2^ of the model. Similar associations were seen in analyses of individual fracture sites (see Supplementary Tables 3, 4 and 5), except that compared to total hip, spine, and wrist fractures the explained variance was greater for hip fracture in HK men and women, and spine fractures for UK men.

## Discussion

This study has shown a number of significant differences between the incidence of fractures and the distribution of factors potentially contributing to fracture risk in two different population cohorts of older individuals, one based in the UK, and the other in Hong Kong. We identified differences in demographic, lifestyle, and biological factors between the two geographically distinct cohorts, and also found that the proportions of fractures at the different bone sites studied differed significantly between the two cohorts. However, the contributions of different factors to fracture risk were broadly similar in multivariable modelling.

Previous research has suggested there are worldwide differences in osteoporotic fracture rates, with the greatest number of fractures occurring in Western populations (Europe, USA, and Australia) [3]. While there has been a recent stabilisation of increases in prevalence of fracture rates in Western populations, the prevalence in developing populations, including Asia, continues to rise [29, 30]. Our results confirm that differences in fracture rates exist between individuals in a UK vs HK population cohort. However, while previous research has specifically identified Hong Kong as a high-risk country for osteoporotic fractures [3], our results showed a lower overall fracture incidence in the HK cohort than in the UK cohort. Our analyses also highlighted variations in the distribution of fractures across hip, spine, and wrist in the two cohorts: The incidence of hip fractures in women of the HK cohort was less than a third of the rate in the UK; spine fracture rates in the HK cohort were approximately half those in the UK, for both men and women; while the rate of wrist fractures in the HK cohort was more than double the UK rate for men and a third higher for women. These fracture incidence rates together with data on the proportions of hip, spine, and wrist, fractures seen in the two cohorts depict a very different scenario for fractures in older individuals in the UK and HK. This has not been fully appreciated by previous studies and thus makes exploration of differences in characteristics of the two cohorts, which may help explain this variation, even more important. It is unclear why the proportions of fractures of different bones differs so much between the cohorts. Differences in body size and composition between the cohorts may be partly responsible, and indeed BMI is an important contributor to fracture risk in both cohorts. We considered whether differences in fracture rates could be due to different age distribution of cohorts, as although we had excluded participants under 65 years old from our analyses, the HK dataset had a greater proportion of older individuals. However, in analyses where the cohort sample was restricted to 65–75 in both cohorts to allow direct comparison, results showed slightly lower fracture rates in both cohorts, particularly in HK, but the differences between HK and UK remained significant. Likewise, we considered whether the different proportions of fractures at different bone sites (hip, spine, and wrist) between cohorts may have been in part driven by age differences, but investigation of this using age-stratified analyses did not support this theory.

Before commencing our statistical analyses we made the decision to include specific demographic, dietary, lifestyle, and biological factors in the multivariable model based on previous knowledge and evidence of what may contribute to fracture risk. It is thus noteworthy that the majority of these factors were not significantly associated with fracture risk in our models. The major driver of fracture risk was age, which in men explained 11.3% of the variance in total fracture risk in each cohort, and in women explained 7.3% and 7.6% in UK and HK cohorts, respectively. BMI was also an important contributor to variance in total fracture risk: 5.1% of variance in total fracture risk was explained by BMI in HK men, and 2.5% in UK women. However, dietary and other variables appeared to contribute very little to fracture risk in our models. In addition to the contribution of each variable to the models, it is important to consider differences in fracture risk for different categories of the variables. In line with previous observations in the UK cohort [31], in women being underweight was associated with much greater fracture risk compared to normal weight individuals, and being overweight or obese was associated with slightly lower risk; in the HK cohort in men underweight was also associated with much greater fracture risk compared to normal weight individuals.

Considering the relative lack of evidence from our models of the importance of lifestyle variables and dietary factors, including calcium and vitamin D, our findings indirectly support the results of recent genome wide association and mendelian randomisation studies which suggest that neither dietary calcium [32] nor vitamin D [32, 33] are causally related to fracture risk, and new data from the China National Fracture Study showing no reduction in fracture risk associated with calcium or vitamin D supplement use[30]. However, within individual populations, lifestyle risk factors still apply, and these may differ in different populations, so that dietary and other recommendations may need to be individualised. This study has shown significant differences between fracture risk in a UK population cohort and HK cohort. This new evidence represents an important advance, and this study is to our knowledge the first comprehensive epidemiological analysis of the difference between fracture risk in the elderly of the UK and HK. While there will inevitably have been different exposures over the life course for individuals in the two cohorts, lifestyle and dietary factors in older age appear to be less important than ageing itself.

### Strengths and Limitations

Our study has a number of strengths. Both the EPIC and Mr and Ms Os cohorts have been extensively reported on in the past, and as such are widely regarded as providing robust datasets for epidemiological analysis and a sample representative of their respective populations [15, 34, 35]. In particular, they both provide a broad range of variables known to be relevant for bone. Where direct matching of variables between the datasets has not been possible, e.g., education and physical activity variables, we have recoded these appropriately to allow comparisons. These cohorts also have the advantage of hospital admission data for fractures, which thus provides much more reliable data for assessing fracture incidence than self-reported fracture data. However, despite the use of hospital admission data, differential admission and diagnostic approaches between HK and UK mean it is possible that we may still have underestimated some fracture incidences, particularly spine and wrist fractures, and the pattern of inaccuracy may differ by sex and country. Length of follow-up differed by approximately 5 years between the two cohorts studied here. We have therefore used standardised fracture rates (per 1000 person-years) to allow direct comparison, but cannot exclude the possibility of inaccuracy introduced into our models by this inconsistency in the datasets. There are also substantial secular changes in fracture incidence rates in both UK and Hong Kong, which are more marked in Hong Kong [36], so differences in fracture rates between the two countries depend on when the comparisons are made; however, the current study was conducted over roughly the same time period mitigating this limitation. Due to fundamental differences in some of the characteristics of participants in the two cohorts, for example the minimal alcohol consumption in the HK cohort compared to the UK, our ability to use an established statistical approach to directly compare the importance of different factors to fracture risk was limited. The R^2^ statistic is generally well understood to give an indication of how much a particular statistical model explains the variance of a dependent variable (in our case, fracture risk). We therefore chose to interpret the significance of contributions of different factors by using an established method [28] to examine the R^2^ statistic for individual factors included a priori in a multivariable model.

Previous observational studies have often focussed on hip fractures alone, due to more readily available epidemiological data than for other sites of osteoporotic fractures. Despite this, fractures at other sites, including the spine and wrist contribute significantly to the global burden of disease, particularly in younger individuals [1]. It is therefore an advantage of our study that data for different fracture sites were available in both UK and HK datasets. Similarly, it is an advantage that we have been able to analyse data for each sex separately, as previous studies have demonstrated distinct sex differences in the relationships between dietary and lifestyle variables with bone measures, including findings from analysis of our cohorts [10, 13]. One of our reasons to study the EPIC and Mr and Ms Os cohorts was due to the similarities in their original data collection methods and the variables available for analysis. We attempted to standardise variables to address any inconsistencies, e.g., in categorisations, but acknowledge the limitations in this, particularly where the distributions between categories were very different between the cohorts. Accurate estimation of dietary nutrient intake is also critical to the validity of the findings of this type of study. The methodology used here of FFQ may not be as precise as dietary intake figures derived from 7 day food diaries [37]. However, 7-day food diary data were not available for both cohorts so we have used FFQ data to match the methodology used to derive nutrient intakes in both datasets. We also acknowledge the limitation that the two cohorts were not recruited at the same time periods and thus there could be a secular effect in the differences in fracture incidence identified.

## Conclusions

This study provides a comprehensive epidemiological analysis of the differences between fracture risk in older individuals of the UK and HK. Significant differences between characteristics of UK and HK study participants were evident, and hip, spine, and wrist fracture risk varied significantly between the cohorts. Despite this, the variables explaining the majority of variance in fracture risk were the same in each cohort, namely age and BMI. More clarity of the reasons for this is required to inform culturally specific interventions and public health recommendations aimed at reducing the burden of osteoporosis in the UK and HK populations.

## Supplementary Information

Below is the link to the electronic supplementary material.

Supplementary file1 (DOCX 20 kb)

Supplementary file2 (DOCX 20 kb)

Supplementary file3 (DOCX 37 kb)

Supplementary file4 (DOCX 37 kb)

Supplementary file5 (DOCX 37 kb)

Supplementary file6 (PPTX 451 kb)

Supplementary file7 (PPTX 481 kb)

Supplementary file8 (PPTX 458 kb)

## Data Availability

The datasets used in our analyses are available to other researchers on request. For further information please contact the corresponding author.

## References

[1] Johnell O, Kanis JA (2006). An estimate of the worldwide prevalence and disability associated with osteoporotic fractures. Osteoporos Int.

[2] Sozen T, Ozisik L, Basaran NC (2017). An overview and management of osteoporosis. Eur J Rheumatol.

[3] Curtis EM, Moon RJ, Harvey NC, Cooper C (2017). The impact of fragility fracture and approaches to osteoporosis risk assessment worldwide. Bone.

[4] Araujo AB, Travison TG, Harris SS, Holick MF, Turner AK, McKinlay JB (2007). Race/ethnic differences in bone mineral density in men. Osteoporos Int.

[5] Cooper C, Campion G, Melton LJ (1992). Hip fractures in the elderly: a world-wide projection. Osteoporos Int.

[6] Gennari C (2001). Calcium and vitamin D nutrition and bone disease of the elderly. Public Health Nutr.

[7] Kong SH, Kim JH, Hong AR, Cho NH, Shin CS (2017). Dietary calcium intake and risk of cardiovascular disease, stroke, and fracture in a population with low calcium intake. Am J Clin Nutr.

[8] Zhao JG, Zeng XT, Wang J, Liu L (2017). Association between calcium or vitamin D supplementation and fracture incidence in community-dwelling older adults: a systematic review and meta-analysis. JAMA.

[9] Kahwati LC, Weber RP, Pan H, Gourlay M, LeBlanc E, Coker-Schwimmer M, Viswanathan M (2018). Vitamin D, calcium, or combined supplementation for the primary prevention of fractures in community-dwelling adults: evidence report and systematic review for the US Preventive Services Task Force. JAMA.

[10] Hayhoe RP, Lentjes MA, Luben RN, Khaw KT, Welch AA (2015). Dietary magnesium and potassium intakes and circulating magnesium are associated with heel bone ultrasound attenuation and osteoporotic fracture risk in the EPIC-Norfolk cohort study. Am J Clin Nutr.

[11] Finck H, Hart AR, Jennings A, Welch AA (2014). Is there a role for vitamin C in preventing osteoporosis and fractures? A review of the potential underlying mechanisms and current epidemiological evidence. Nutr Res Rev.

[12] Li Y, Li D, Ma CY, Liu CY, Hui D, Wen ZM, Peng LP (2012). Consumption of, and factors influencing consumption of, fruit and vegetables among elderly Chinese people. Nutrition.

[13] Chan R, Woo J, Leung J (2011). Effects of food groups and dietary nutrients on bone loss in elderly Chinese population. J Nutr Health Aging.

[14] Chan R, Chan D, Woo J, Ohlsson C, Mellstrom D, Kwok T, Leung PC (2012). Not all elderly people benefit from vitamin D supplementation with respect to physical function: results from the Osteoporotic Fractures in Men Study, Hong Kong. J Am Geriatr Soc.

[15] Day N, Oakes S, Luben R, Khaw KT, Bingham S, Welch A, Wareham N (1999). EPIC-Norfolk: study design and characteristics of the cohort. European Prospective Investigation of Cancer. Br J Cancer.

[16] Wong SY, Kwok T, Woo J, Lynn H, Griffith JF, Leung J, Tang YY, Leung PC (2005). Bone mineral density and the risk of peripheral arterial disease in men and women: results from Mr. and Ms Os. Hong Kong Osteoporos Int.

[17] Census and Statistics Department (2006). Population Bycensus Thematic Report: Older Persons.

[18] Bingham SA, Welch AA, McTaggart A, Mulligan AA, Runswick SA, Luben R, Oakes S, Khaw KT, Wareham N, Day NE (2001). Nutritional methods in the European Prospective Investigation of Cancer in Norfolk. Public Health Nutr.

[19] Welch AA, Luben R, Khaw KT, Bingham SA (2005). The CAFE computer program for nutritional analysis of the EPIC-Norfolk food frequency questionnaire and identification of extreme nutrient values. J Hum Nutr Diet.

[20] Woo J, Leung SSF, Ho SC, Lam TH, Janus ED (1997). A food frequency questionnaire for use in the Chinese population in Hong Kong:description and examination of validity. Nutr Res.

[21] Paul AA, Southgate DAT (1978). McCance & Widdowson’s: The Composition of Foods.

[22] Yang YWG, Pan X (2002). China Food Composition.

[23] Moayyeri A, Kaptoge S, Dalzell N, Bingham S, Luben RN, Wareham NJ, Reeve J, Khaw KT (2009). Is QUS or DXA better for predicting the 10-year absolute risk of fracture?. J Bone Miner Res.

[24] Washburn RA, Smith KW, Jette AM, Janney CA (1993). The Physical Activity Scale for the Elderly (PASE): development and evaluation. J Clin Epidemiol.

[25] Wareham NJ, Jakes RW, Rennie KL, Schuit J, Mitchell J, Hennings S, Day NE (2003). Validity and repeatability of a simple index derived from the short physical activity questionnaire used in the European Prospective Investigation into Cancer and Nutrition (EPIC) study. Public Health Nutr.

[26] Welch A, Camus J, Dalzell N, Oakes S, Reeve J, Khaw KT (2004). Broadband ultrasound attenuation (BUA) of the heel bone and its correlates in men and women in the EPIC-Norfolk cohort: a cross-sectional population-based study. Osteoporos Int.

[27] Jakes RW, Khaw K, Day NE, Bingham S, Welch A, Oakes S, Luben R, Dalzell N, Reeve J, Wareham NJ (2001). Patterns of physical activity and ultrasound attenuation by heel bone among Norfolk cohort of European Prospective Investigation of Cancer (EPIC Norfolk): population based study. BMJ.

[28] Heller G (2012). A measure of explained risk in the proportional hazards model. Biostatistics.

[29] Cooper C, Cole ZA, Holroyd CR, Earl SC, Harvey NC, Dennison EM, Melton LJ, Cummings SR, Kanis JA (2011). Secular trends in the incidence of hip and other osteoporotic fractures. Osteoporos Int.

[30] Lv H, Chen W, Zhang T (2020). Traumatic fractures in China from 2012 to 2014: a National Survey of 512,187 individuals. Osteoporos Int.

[31] Moayyeri A, Luben RN, Bingham SA, Welch AA, Wareham NJ, Khaw KT (2008). Measured height loss predicts fractures in middle-aged and older men and women: the EPIC-Norfolk prospective population study. J Bone Miner Res.

[32] Trajanoska K, Morris JA, Oei L (2018). Assessment of the genetic and clinical determinants of fracture risk: genome wide association and mendelian randomisation study. BMJ.

[33] Larsson SC, Melhus H, Michaelsson K (2018). Circulating serum 25-hydroxyvitamin D levels and bone mineral density: Mendelian randomization study. J Bone Miner Res.

[34] Lau EM, Leung PC, Kwok T, Woo J, Lynn H, Orwoll E, Cummings S, Cauley J (2006). The determinants of bone mineral density in Chinese men-results from Mr. Os (Hong Kong), the first cohort study on osteoporosis in Asian men. Osteoporos Int.

[35] Khoo CC, Woo J, Leung PC, Kwok A, Kwok T (2011). Determinants of bone mineral density in older postmenopausal Chinese women. Climacteric.

[36] Lau EM, Cooper C, Wickham C, Donnan S, Barker DJ (1990). Hip fracture in Hong Kong and Britain. Int J Epidemiol.

[37] Lentjes MA, McTaggart A, Mulligan AA, Powell NA, Parry-Smith D, Luben RN, Bhaniani A, Welch AA, Khaw KT (2013). Dietary intake measurement using 7 d diet diaries in British men and women in the European Prospective Investigation into Cancer-Norfolk study: a focus on methodological issues. Br J Nutr.

