# Supplementary material for: Fracture Incidence and the Relevance of Dietary and Lifestyle Factors Differ in the United Kingdom and Hong Kong: An International Comparison of Longitudinal Cohort Study Data
Source: Calcif Tissue Int. 2021 Jun 3;109(5):563–76. doi: 10.1007/s00223-021-00870-z (PMC8484188; doi:10.1007/s00223-021-00870-z)
Supplement: Supplementary file 1 — Supplementary file1 (DOCX 20 kb) [file 223_2021_870_MOESM1_ESM.docx]

**Supplementary Table 1.** Comparison of incident fracture risks* in men and women of the UK and HK cohorts.

|  |  | **UK** |  |  |  |  | **HK** |  |  |  |  | **P value** |
| --- | --- | --- | --- | --- | --- | --- | --- | --- | --- | --- | --- | --- |
| **Sex** | **Fracture site** | **Person-years** | **Failure** | **Rate (per 1000 person-years)** | **95% CI** |  | **Person-years** | **Failure** | **Rate (per 1000 person-years)** | **95% CI** |  |  |
| **Men** | **Total hip, spine and wrist** | 50282.37 | 299 | 5.95 | 5.31 | 6.66 | 23107.50 | 124 | 5.37 | 4.50 | 6.40 | 0.337 |
|  | **Hip** | 50739.77 | 177 | 3.49 | 3.01 | 4.04 | 23564.18 | 62 | 2.63 | 2.05 | 3.37 | 0.053 |
|  | **Spine** | 50839.88 | 107 | 2.10 | 1.74 | 2.54 | 23664.47 | 26 | 1.10 | 0.75 | 1.61 | <0.001 |
|  | **Wrist** | 51143.05 | 35 | 0.68 | 0.49 | 0.95 | 23501.06 | 42 | 1.79 | 1.32 | 2.42 | 0.002 |
| **Women** | **Total hip, spine and wrist** | 61554.82 | 843 | 13.70 | 12.80 | 14.65 | 23397.84 | 205 | 8.76 | 7.64 | 10.05 | <0.001 |
|  | **Hip** | 63345.30 | 522 | 8.24 | 7.56 | 8.98 | 24707.02 | 66 | 2.67 | 2.10 | 3.40 | <0.001 |
|  | **Spine** | 64231.61 | 266 | 4.14 | 3.67 | 4.67 | 24746.80 | 48 | 1.94 | 1.46 | 2.57 | 0.015 |
|  | **Wrist** | 64460.00 | 201 | 3.12 | 2.72 | 3.58 | 24180.08 | 102 | 4.22 | 3.47 | 5.12 | <0.001 |

*count the occurrence of 1^st^ fracture only
