# Supplementary material for: Fracture Incidence and the Relevance of Dietary and Lifestyle Factors Differ in the United Kingdom and Hong Kong: An International Comparison of Longitudinal Cohort Study Data
Source: Calcif Tissue Int. 2021 Jun 3;109(5):563–76. doi: 10.1007/s00223-021-00870-z (PMC8484188; doi:10.1007/s00223-021-00870-z)
Supplement: Supplementary file 2 — Supplementary file2 (DOCX 20 kb) [file 223_2021_870_MOESM2_ESM.docx]

**Supplementary Table 2.** Comparison of incident fracture risks* in men 65 to 75y only

| **Sex** | **Fracture site** | **UK** |  |  |  |  | **HK** |  |  |  |  | **P value** |
| --- | --- | --- | --- | --- | --- | --- | --- | --- | --- | --- | --- | --- |
|  |  | **Person-years** | **Failure** | **Rate (per 1000 person-years)** | **95% CI** |  | **Person-years** | **Failure** | **Rate (per 1000 person-years)** | **95% CI** |  |  |
| **Men** | **Total hip, spine and wrist** | 46441.76 | 268 | 5.77 | 5.12 | 6.50 | 18398.86 | 74 | 4.02 | 3.2 | 5.05 | 0.005 |
|  | **Hip** | 46867.74 | 162 | 3.46 | 2.96 | 4.03 | 18736.68 | 29 | 1.55 | 1.08 | 2.23 | <0.001 |
|  | **Spine** | 46947.49 | 97 | 2.07 | 1.69 | 2.52 | 18769.14 | 15 | 0.80 | 0.48 | 1.33 | <0.001 |
|  | **Wrist** | 47253.51 | 26 | 0.55 | 0.37 | 0.81 | 18638.18 | 33 | 1.77 | 1.26 | 2.49 | <0.001 |
| **Women** | **Total hip, spine and wrist** | 57009.00 | 757 | 13.28 | 12.37 | 14.26 | 17473.24 | 121 | 6.92 | 5.79 | 8.28 | <0.001 |
|  | **Hip** | 58629.18 | 469 | 8.00 | 7.31 | 8.76 | 18436.79 | 25 | 1.36 | 0.92 | 2.01 | <0.001 |
|  | **Spine** | 59435.57 | 238 | 4.00 | 3.53 | 4.55 | 18386.53 | 28 | 1.52 | 1.05 | 2.21 | <0.001 |
|  | **Wrist** | 59607.73 | 184 | 3.09 | 2.67 | 3.57 | 17904.79 | 73 | 4.08 | 3.24 | 5.13 | 0.048 |

*count the occurrence of 1^st^ fracture only
