# Supplementary material for: Fracture Incidence and the Relevance of Dietary and Lifestyle Factors Differ in the United Kingdom and Hong Kong: An International Comparison of Longitudinal Cohort Study Data
Source: Calcif Tissue Int. 2021 Jun 3;109(5):563–76. doi: 10.1007/s00223-021-00870-z (PMC8484188; doi:10.1007/s00223-021-00870-z)
Supplement: Supplementary file 3 — Supplementary file3 (DOCX 37 kb) [file 223_2021_870_MOESM3_ESM.docx]

**Supplementary Table 3.** Multivariate Cox regression results linking various contributory factors to the risk of hip fractures in both study sites

|  | **UK men** |  |  |  |  | **HK men** | |  |  |  | **UK women** | |  |  |  | **HK women** | |  |  |  |
| --- | --- | --- | --- | --- | --- | --- | --- | --- | --- | --- | --- | --- | --- | --- | --- | --- | --- | --- | --- | --- |
| **Characteristic** | **HR*^1^*** | **95% CI*^1^*** | **p-value** | **omnibus p** | **R^2^** | **HR*^1^*** | **95% CI*^1^*** | **p-value** | **omnibus p** | **R^2^** | **HR*^1^*** | **95% CI*^1^*** | **p-value** | **omnibus p** | **R^2^** | **HR*^1^*** | **95% CI*^1^*** | **p-value** | **omnibus p** | **R^2^** |
| Age group |  |  |  | <0.001 | 0.1023 |  |  |  | <0.001 | 0.196 |  |  |  | <0.001 | 0.0824 |  |  |  | <0.001 | 0.3216 |
| <70 | 1.00 | — |  |  |  | 1.00 | — |  |  |  | 1.00 | — |  |  |  | 1.00 | — |  |  |  |
| 70 to <75 | 2.15 | 1.57, 2.94 | <0.001 |  |  | 2.22 | 0.97, 5.10 | 0.060 |  |  | 1.90 | 1.58, 2.29 | <0.001 |  |  | 3.29 | 1.30, 8.35 | 0.012 |  |  |
| 75 to <80 | 2.10 | 1.20, 3.68 | 0.010 |  |  | 4.29 | 1.87, 9.85 | <0.001 |  |  | 2.25 | 1.65, 3.06 | <0.001 |  |  | 6.61 | 2.66, 16.4 | <0.001 |  |  |
| ≥80 HK |  |  |  |  |  | 7.25 | 2.97, 17.7 | <0.001 |  |  |  |  |  |  |  | 10.30 | 3.95, 26.8 | <0.001 |  |  |
| BMI categories (kg/m^2^) |  |  |  | 0.166 | 0.0227 |  |  |  | 0.003 | 0.096 |  |  |  | <0.001 | 0.0441 |  |  |  | 0.284 | 0.0271 |
| <18.5 | 3.22 | 0.44, 23.6 | 0.249 |  |  | 2.48 | 1.19, 5.16 | 0.015 |  |  | 2.78 | 1.42, 5.45 | 0.003 |  |  | 1.86 | 0.79, 4.40 | 0.157 |  |  |
| 18.5 to <25 UK; 18.5 to <23 HK | 1.00 | — |  |  |  | 1.00 | — |  |  |  | 1.00 | — |  |  |  | 1.00 | — |  |  |  |
| 25 to <30 UK; 23 to <25 HK | 0.73 | 0.52, 1.01 | 0.061 |  |  | 0.46 | 0.22, 0.99 | 0.046 |  |  | 0.67 | 0.55, 0.81 | <0.001 |  |  | 1.26 | 0.69, 2.32 | 0.454 |  |  |
| ≥30 UK; ≥25 HK | 0.94 | 0.59, 1.51 | 0.806 |  |  | 0.61 | 0.32, 1.17 | 0.135 |  |  | 0.64 | 0.49, 0.83 | <0.001 |  |  | 0.79 | 0.42, 1.51 | 0.481 |  |  |
| Physical activity level |  |  |  | 0.460 | 0.0107 |  |  |  | 0.110 | 0.043 |  |  |  | 0.065 | 0.0107 |  |  |  | 0.729 | 0.0057 |
| Inactive | 1.00 | — |  |  |  | 1.00 | — |  |  |  | 1.00 | — |  |  |  | 1.00 | — |  |  |  |
| Moderately inactive | 0.87 | 0.60, 1.27 | 0.478 |  |  | 1.18 | 0.68, 2.04 | 0.548 |  |  | 0.80 | 0.65, 0.98 | 0.031 |  |  | 0.77 | 0.39, 1.53 | 0.452 |  |  |
| Moderately active UK; Active/moderately active HK | 0.71 | 0.46, 1.11 | 0.135 |  |  | 0.45 | 0.17, 1.19 | 0.107 |  |  | 0.76 | 0.58, 0.99 | 0.045 |  |  | 1.05 | 0.37, 2.96 | 0.934 |  |  |
| Active UK | 0.98 | 0.64, 1.52 | 0.937 |  |  |  |  |  |  |  | 0.78 | 0.56, 1.10 | 0.159 |  |  |  |  |  |  |  |
| Smoking status |  |  |  | 0.671 | 0.0007 |  |  |  | 0.376 | 0.0063 |  |  |  | 0.948 | 0.0000 |  |  |  | 0.908 | 0.0001 |
| Never smoked | 1.00 | — |  |  |  | 1.00 | — |  |  |  | 1.00 | — |  |  |  | 1.00 | — |  |  |  |
| Current or former smoker | 1.08 | 0.76, 1.53 | 0.673 |  |  | 1.28 | 0.73, 2.25 | 0.383 |  |  | 0.99 | 0.83, 1.19 | 0.948 |  |  | 0.96 | 0.45, 2.04 | 0.908 |  |  |
| Family Hx of osteoporosis |  |  |  | 0.156 | 0.0068 |  |  |  | 0.576 | 0.0031 |  |  |  | 0.491 | 0.0007 |  |  |  | 0.159 | 0.0159 |
| No | 1.00 | — |  |  |  | 1.00 | — |  |  |  | 1.00 | — |  |  |  | 1.00 | — |  |  |  |
| Yes | 1.93 | 0.84, 4.39 | 0.119 |  |  | 0.68 | 0.17, 2.82 | 0.598 |  |  | 1.18 | 0.75, 1.84 | 0.481 |  |  | 2.09 | 0.82, 5.33 | 0.122 |  |  |
| Education |  |  |  | 0.517 | 0.0052 |  |  |  | 0.927 | 0.0013 |  |  |  | 0.807 | 0.0006 |  |  |  | 0.331 | 0.0180 |
| None/pre-secondary | 1.00 | — |  |  |  | 1.00 | — |  |  |  | 1.00 | — |  |  |  | 1.00 | — |  |  |  |
| Secondary/further education | 1.09 | 0.79, 1.50 | 0.593 |  |  | 1.05 | 0.58, 1.91 | 0.862 |  |  | 0.98 | 0.81, 1.18 | 0.815 |  |  | 0.88 | 0.37, 2.08 | 0.773 |  |  |
| Higher education | 0.81 | 0.47, 1.40 | 0.457 |  |  | 0.88 | 0.38, 2.03 | 0.765 |  |  | 1.11 | 0.77, 1.58 | 0.582 |  |  | 1.99 | 0.82, 4.80 | 0.127 |  |  |
| Dietary Ca meeting RNI |  |  |  | 0.909 | 0.0001 |  |  |  | 0.114 | 0.0192 |  |  |  | 0.289 | 0.0016 |  |  |  | 0.711 | 0.0012 |
| No | 1.00 | — |  |  |  | 1.00 | — |  |  |  | 1.00 | — |  |  |  | 1.00 | — |  |  |  |
| Yes | 0.97 | 0.60, 1.57 | 0.908 |  |  | 1.54 | 0.91, 2.60 | 0.111 |  |  | 0.88 | 0.69, 1.11 | 0.282 |  |  | 0.89 | 0.49, 1.63 | 0.713 |  |  |
| Dietary vitamin D intake (ug/1000 kcal) | 0.99 | 0.82, 1.20 | 0.917 | 0.917 | 0.0000 | 1.45 | 0.76, 2.77 | 0.263 | 0.318 | 0.0051 | 1.05 | 0.95, 1.16 | 0.306 | 0.310 | 0.0014 | 1.96 | 0.76, 5.04 | 0.161 | 0.185 | 0.0120 |
| Vegetable consumption (g/100 kcal/d) | 0.99 | 0.96, 1.02 | 0.363 | 0.355 | 0.0042 | 1.03 | 1.00, 1.05 | 0.022 | 0.044 | 0.0261 | 1.00 | 0.99, 1.01 | 0.890 | 0.890 | 0.0000 | 1.01 | 0.98, 1.03 | 0.538 | 0.561 | 0.0027 |
| Fruit consumption (g/100 kcal/d) | 1.01 | 0.99, 1.03 | 0.323 | 0.333 | 0.0048 | 0.97 | 0.94, 1.01 | 0.161 | 0.137 | 0.0202 | 1.00 | 0.99, 1.01 | 0.824 | 0.824 | 0.0001 | 0.99 | 0.96, 1.02 | 0.490 | 0.481 | 0.0043 |
| Ethanol consumption (units/d) |  |  |  | 0.728 | 0.0027 |  |  |  | 0.809 | 0.0005 |  |  |  | 0.012 | 0.0147 |  |  |  |  |  |
| None | 1.00 | — |  |  |  | 1.00 | — |  |  |  | 1.00 | — |  |  |  |  |  |  |  |  |
| >0 to <2 UK; >0 HK | 0.96 | 0.64, 1.44 | 0.842 |  |  | 0.92 | 0.48, 1.77 | 0.810 |  |  | 0.92 | 0.76, 1.11 | 0.361 |  |  |  |  |  |  |  |
| ≥2 UK | 1.12 | 0.68, 1.83 | 0.652 |  |  |  |  |  |  |  | 0.54 | 0.34, 0.83 | 0.006 |  |  |  |  |  |  |  |
| Use of Ca supplement |  |  |  | 0.443 | 0.0021 |  |  |  | 0.664 | 0.0015 |  |  |  | 0.643 | 0.0003 |  |  |  |  |  |
| No | 1.00 | — |  |  |  | 1.00 | — |  |  |  | 1.00 | — |  |  |  | 1.00 | — |  |  |  |
| Yes | 1.52 | 0.56, 4.14 | 0.415 |  |  | 0.82 | 0.32, 2.07 | 0.673 |  |  | 1.11 | 0.72, 1.69 | 0.638 |  |  | 0.98 | 0.52, 1.83 | 0.942 | 0.942 | 0.0000 |
| HRT use |  |  |  |  |  |  |  |  |  |  |  |  |  | 0.804 | 0.0001 |  |  |  |  |  |
| Never |  |  |  |  |  |  |  |  |  |  | 1.00 | — |  |  |  |  |  |  |  |  |
| Past/current |  |  |  |  |  |  |  |  |  |  | 1.03 | 0.80, 1.34 | 0.805 |  |  |  |  |  |  |  |
| *^1^* HR = Hazard Ratio, CI = Confidence Interval | | | |  |  |  | | |  |  |  | | |  |  |  | | |  |  |
