# Supplementary material for: Fracture Incidence and the Relevance of Dietary and Lifestyle Factors Differ in the United Kingdom and Hong Kong: An International Comparison of Longitudinal Cohort Study Data
Source: Calcif Tissue Int. 2021 Jun 3;109(5):563–76. doi: 10.1007/s00223-021-00870-z (PMC8484188; doi:10.1007/s00223-021-00870-z)
Supplement: Supplementary file 4 — Supplementary file4 (DOCX 37 kb) [file 223_2021_870_MOESM4_ESM.docx]

**Supplementary Table 4.** Multivariate Cox regression results linking various contributory factors to the risk of spine fractures in both study sites

|  | **UK men** |  |  |  |  | **HK men** | |  |  |  | **UK women** | |  |  |  | **HK women** | |  |  |  |
| --- | --- | --- | --- | --- | --- | --- | --- | --- | --- | --- | --- | --- | --- | --- | --- | --- | --- | --- | --- | --- |
| **Characteristic** | **HR*^1^*** | **95% CI*^1^*** | **p-value** | **omnibus p** | **R^2^** | **HR*^1^*** | **95% CI*^1^*** | **p-value** | **omnibus p** | **R^2^** | **HR*^1^*** | **95% CI*^1^*** | **p-value** | **omnibus p** | **R^2^** | **HR*^1^*** | **95% CI*^1^*** | **p-value** | **omnibus p** | **R^2^** |
| Age group |  |  |  | <0.001 | 0.3151 |  |  |  | 0.508 | 0.0397 |  |  |  | 0.001 | 0.0524 |  |  |  | 0.167 | 0.0364 |
| <70 | 1.00 | — |  |  |  | 1.00 | — |  |  |  | 1.00 | — |  |  |  | 1.00 | — |  |  |  |
| 70 to <75 | 2.87 | 1.29, 6.38 | 0.009 |  |  | 1.34 | 0.64, 2.84 | 0.439 |  |  | 1.74 | 1.30, 2.34 | <0.001 |  |  | 1.59 | 0.97, 2.61 | 0.065 |  |  |
| 75 to <80 | 10.00 | 3.96, 25.2 | <0.001 |  |  | 1.81 | 0.78, 4.21 | 0.166 |  |  | 1.56 | 0.92, 2.65 | 0.102 |  |  | 1.40 | 0.81, 2.44 | 0.232 |  |  |
| ≥80 HK |  |  |  |  |  | 0.85 | 0.19, 3.85 | 0.831 |  |  |  |  |  |  |  | 0.87 | 0.38, 1.98 | 0.744 |  |  |
| BMI categories (kg/m^2^) |  |  |  | 0.723 | 0.0115 |  |  |  | 0.400 | 0.0434 |  |  |  | 0.061 | 0.0287 |  |  |  | 0.239 | 0.0283 |
| <18.5 |  |  |  |  |  | 1.71 | 0.48, 6.06 | 0.405 |  |  | 0.59 | 0.08, 4.24 | 0.598 |  |  | 2.04 | 0.93, 4.45 | 0.074 |  |  |
| 18.5 to <25 UK; 18.5 to <23 HK | 1.00 | — |  |  |  | 1.00 | — |  |  |  | 1.00 | — |  |  |  | 1.00 | — |  |  |  |
| 25 to <30 UK; 23 to <25 HK | 0.89 | 0.43, 1.86 | 0.761 |  |  | 0.90 | 0.38, 2.12 | 0.812 |  |  | 0.79 | 0.59, 1.07 | 0.124 |  |  | 0.89 | 0.51, 1.56 | 0.688 |  |  |
| ≥30 UK; ≥25 HK | 0.60 | 0.17, 2.18 | 0.442 |  |  | 1.62 | 0.78, 3.36 | 0.195 |  |  | 0.56 | 0.36, 0.88 | 0.012 |  |  | 1.23 | 0.77, 1.96 | 0.381 |  |  |
| Physical activity level |  |  |  | 0.773 | 0.0149 |  |  |  | 0.535 | 0.0192 |  |  |  | 0.402 | 0.0106 |  |  |  | 0.019 | 0.0527 |
| Inactive | 1.00 | — |  |  |  | 1.00 | — |  |  |  | 1.00 | — |  |  |  | 1.00 | — |  |  |  |
| Moderately inactive | 1.09 | 0.45, 2.62 | 0.846 |  |  | 1.48 | 0.74, 2.95 | 0.263 |  |  | 0.81 | 0.58, 1.13 | 0.205 |  |  | 1.80 | 1.18, 2.76 | 0.007 |  |  |
| Moderately active UK; Active/moderately active HK | 1.26 | 0.49, 3.23 | 0.627 |  |  | 1.25 | 0.53, 2.96 | 0.608 |  |  | 1.09 | 0.73, 1.63 | 0.659 |  |  | 0.75 | 0.30, 1.89 | 0.548 |  |  |
| Active UK | 1.65 | 0.64, 4.24 | 0.297 |  |  |  |  |  |  |  | 0.79 | 0.45, 1.39 | 0.406 |  |  |  |  |  |  |  |
| Smoking status |  |  |  | 0.667 | 0.0026 |  |  |  | 0.353 | 0.0135 |  |  |  | 0.629 | 0.0009 |  |  |  | 0.183 | 0.0123 |
| Never smoked | 1.00 | — |  |  |  | 1.00 | — |  |  |  | 1.00 | — |  |  |  | 1.00 | — |  |  |  |
| Current or former smoker | 0.85 | 0.40, 1.79 | 0.663 |  |  | 0.74 | 0.39, 1.39 | 0.350 |  |  | 0.93 | 0.70, 1.24 | 0.630 |  |  | 1.53 | 0.84, 2.78 | 0.162 |  |  |
| Family Hx of osteoporosis |  |  |  | 0.602 | 0.0036 |  |  |  | 0.306 | 0.0136 |  |  |  | 0.100 | 0.0135 |  |  |  | 0.510 | 0.0039 |
| No | 1.00 | — |  |  |  | 1.00 | — |  |  |  | 1.00 | — |  |  |  | 1.00 | — |  |  |  |
| Yes | 1.79 | 0.24, 13.3 | 0.570 |  |  | 1.80 | 0.63, 5.09 | 0.269 |  |  | 0.43 | 0.14, 1.36 | 0.153 |  |  | 0.69 | 0.22, 2.20 | 0.533 |  |  |
| Education |  |  |  | 0.374 | 0.0296 |  |  |  | 0.043 | 0.1082 |  |  |  | 0.702 | 0.0027 |  |  |  | 0.505 | 0.0109 |
| None/pre-secondary | 1.00 | — |  |  |  | 1.00 | — |  |  |  | 1.00 | — |  |  |  | 1.00 | — |  |  |  |
| Secondary/further education | 0.62 | 0.30, 1.27 | 0.193 |  |  | 0.44 | 0.17, 1.16 | 0.098 |  |  | 1.07 | 0.80, 1.44 | 0.652 |  |  | 0.66 | 0.32, 1.38 | 0.270 |  |  |
| Higher education | 0.58 | 0.16, 2.01 | 0.388 |  |  | 1.66 | 0.75, 3.66 | 0.213 |  |  | 0.82 | 0.43, 1.58 | 0.560 |  |  | 0.90 | 0.36, 2.27 | 0.819 |  |  |
| Dietary Ca meeting RNI |  |  |  | 0.267 | 0.0120 |  |  |  | 0.876 | 0.0004 |  |  |  | 0.016 | 0.0200 |  |  |  | 0.320 | 0.0075 |
| No | 1.00 | — |  |  |  | 1.00 | — |  |  |  | 1.00 | — |  |  |  | 1.00 | — |  |  |  |
| Yes | 2.08 | 0.49, 8.75 | 0.318 |  |  | 0.95 | 0.48, 1.86 | 0.876 |  |  | 0.64 | 0.45, 0.90 | 0.012 |  |  | 0.79 | 0.49, 1.27 | 0.329 |  |  |
| Dietary vitamin D intake (ug/1000 kcal) | 1.33 | 0.92, 1.92 | 0.134 | 0.154 | 0.0276 | 0.34 | 0.04, 2.78 | 0.317 | 0.259 | 0.0321 | 1.00 | 0.85, 1.18 | 0.961 | 0.961 | 0.0000 | 1.09 | 0.43, 2.75 | 0.850 | 0.851 | 0.0003 |
| Vegetable consumption (g/100 kcal/d) | 1.01 | 0.95, 1.08 | 0.668 | 0.673 | 0.0031 | 0.99 | 0.95, 1.04 | 0.684 | 0.675 | 0.0030 | 1.01 | 0.99, 1.03 | 0.227 | 0.239 | 0.0053 | 1.01 | 1.00, 1.03 | 0.125 | 0.161 | 0.0144 |
| Fruit consumption (g/100 kcal/d) | 1.00 | 0.95, 1.05 | 0.988 | 0.988 | 0.0000 | 0.99 | 0.95, 1.03 | 0.494 | 0.473 | 0.0080 | 1.00 | 0.98, 1.01 | 0.547 | 0.542 | 0.0016 | 1.01 | 0.99, 1.03 | 0.266 | 0.282 | 0.0075 |
| Ethanol consumption (units/d) |  |  |  | 0.732 | 0.0088 |  |  |  | 0.390 | 0.0112 |  |  |  | 0.169 | 0.0147 |  |  |  | 0.239 | 0.0131 |
| None | 1.00 | — |  |  |  | 1.00 | — |  |  |  | 1.00 | — |  |  |  | 1.00 | — |  |  |  |
| >0 to <2 UK; >0 HK | 0.71 | 0.31, 1.64 | 0.423 |  |  | 1.36 | 0.68, 2.70 | 0.380 |  |  | 0.83 | 0.62, 1.12 | 0.228 |  |  | 0.37 | 0.05, 2.66 | 0.322 |  |  |
| ≥2 UK | 0.81 | 0.27, 2.40 | 0.699 |  |  |  |  |  |  |  | 0.56 | 0.28, 1.10 | 0.093 |  |  |  |  |  |  |  |
| Use of Ca supplement |  |  |  |  |  |  |  |  | 0.642 | 0.0031 |  |  |  | 0.993 | 0.0000 |  |  |  | 0.666 | 0.0013 |
| No |  |  |  |  |  | 1.00 | — |  |  |  | 1.00 | — |  |  |  | 1.00 | — |  |  |  |
| Yes |  |  |  |  |  | 1.26 | 0.49, 3.26 | 0.632 |  |  | 1.00 | 0.49, 2.05 | 0.993 |  |  | 1.12 | 0.67, 1.86 | 0.662 |  |  |
| HRT use |  |  |  |  |  |  |  |  |  |  |  |  |  | 0.256 | 0.0052 |  |  |  | 0.574 | 0.0029 |
| Never |  |  |  |  |  |  |  |  |  |  | 1.00 | — |  |  |  | 1.00 | — |  |  |  |
| Past/current |  |  |  |  |  |  |  |  |  |  | 1.29 | 0.82, 2.02 | 0.271 |  |  | 1.47 | 0.35, 6.17 | 0.596 |  |  |
