# Supplementary material for: Fracture Incidence and the Relevance of Dietary and Lifestyle Factors Differ in the United Kingdom and Hong Kong: An International Comparison of Longitudinal Cohort Study Data
Source: Calcif Tissue Int. 2021 Jun 3;109(5):563–76. doi: 10.1007/s00223-021-00870-z (PMC8484188; doi:10.1007/s00223-021-00870-z)
Supplement: Supplementary file 5 — Supplementary file5 (DOCX 37 kb) [file 223_2021_870_MOESM5_ESM.docx]

**Supplementary Table 5.** Multivariate Cox regression results linking various contributory factors to the risk of wrist fractures in both study sites

|  | **UK men** |  |  |  |  | **HK men** | |  |  |  | **UK women** | |  |  |  | **HK women** | |  |  |  |
| --- | --- | --- | --- | --- | --- | --- | --- | --- | --- | --- | --- | --- | --- | --- | --- | --- | --- | --- | --- | --- |
| **Characteristic** | **HR*^1^*** | **95% CI*^1^*** | **p-value** | **omnibus p** | **R^2^** | **HR*^1^*** | **95% CI*^1^*** | **p-value** | **omnibus p** | **R^2^** | **HR*^1^*** | **95% CI*^1^*** | **p-value** | **omnibus p** | **R^2^** | **HR*^1^*** | **95% CI*^1^*** | **p-value** | **omnibus p** | **R^2^** |
| Age group |  |  |  | 0.037 | 0.0497 |  |  |  | 0.680 | 0.0303 |  |  |  | 0.001 | 0.0428 |  |  |  | 0.176 | 0.0675 |
| <70 | 1.00 | — |  |  |  | 1.00 | — |  |  |  | 1.00 | — |  |  |  | 1.00 | — |  |  |  |
| 70 to <75 | 1.61 | 1.08, 2.41 | 0.020 |  |  | 1.32 | 0.46, 3.74 | 0.603 |  |  | 1.47 | 1.14, 1.91 | 0.003 |  |  | 1.15 | 0.52, 2.55 | 0.732 |  |  |
| 75 to <80 | 1.85 | 0.93, 3.67 | 0.080 |  |  | 1.62 | 0.53, 4.96 | 0.398 |  |  | 1.95 | 1.28, 2.97 | 0.002 |  |  | 1.79 | 0.81, 3.95 | 0.149 |  |  |
| ≥80 HK |  |  |  |  |  | 2.18 | 0.59, 8.05 | 0.241 |  |  |  |  |  |  |  | 2.52 | 1.02, 6.21 | 0.046 |  |  |
| BMI categories (kg/m^2^) |  |  |  | 0.781 | 0.0038 |  |  |  | 0.416 | 0.0543 |  |  |  | 0.370 | 0.0101 |  |  |  | 0.898 | 0.0078 |
| <18.5 |  |  |  |  |  | 1.98 | 0.53, 7.33 | 0.307 |  |  | 1.99 | 0.63, 6.33 | 0.243 |  |  | 0.79 | 0.18, 3.49 | 0.760 |  |  |
| 18.5 to <25 UK; 18.5 to <23 HK | 1.00 | — |  |  |  | 1.00 | — |  |  |  | 1.00 | — |  |  |  | 1.00 | — |  |  |  |
| 25 to <30 UK; 23 to <25 HK | 0.88 | 0.58, 1.35 | 0.560 |  |  | 1.13 | 0.44, 2.91 | 0.794 |  |  | 1.04 | 0.79, 1.35 | 0.795 |  |  | 1.25 | 0.60, 2.62 | 0.549 |  |  |
| ≥30 UK; ≥25 HK | 0.82 | 0.43, 1.56 | 0.544 |  |  | 0.59 | 0.20, 1.74 | 0.340 |  |  | 0.80 | 0.55, 1.17 | 0.260 |  |  | 1.13 | 0.57, 2.27 | 0.724 |  |  |
| Physical activity level |  |  |  | 0.863 | 0.0053 |  |  |  | 0.622 | 0.0198 |  |  |  | 0.533 | 0.0064 |  |  |  | 0.095 | 0.0857 |
| Inactive | 1.00 | — |  |  |  | 1.00 | — |  |  |  | 1.00 | — |  |  |  | 1.00 | — |  |  |  |
| Moderately inactive | 1.20 | 0.75, 1.93 | 0.441 |  |  | 0.64 | 0.25, 1.67 | 0.364 |  |  | 1.14 | 0.86, 1.50 | 0.370 |  |  | 0.37 | 0.13, 1.03 | 0.057 |  |  |
| Moderately active UK; Active/moderately active HK | 0.98 | 0.57, 1.70 | 0.945 |  |  | 0.74 | 0.24, 2.26 | 0.593 |  |  | 0.87 | 0.59, 1.28 | 0.475 |  |  | 0.82 | 0.25, 2.70 | 0.746 |  |  |
| Active UK | 1.09 | 0.61, 1.96 | 0.767 |  |  |  |  |  |  |  | 1.14 | 0.72, 1.79 | 0.579 |  |  |  |  |  |  |  |
| Smoking status |  |  |  | 0.277 | 0.0088 |  |  |  | 0.005 | 0.2101 |  |  |  | 0.062 | 0.0106 |  |  |  | 0.827 | 0.0006 |
| Never smoked | 1.00 | — |  |  |  | 1.00 | — |  |  |  | 1.00 | — |  |  |  | 1.00 | — |  |  |  |
| Current or former smoker | 1.29 | 0.81, 2.06 | 0.287 |  |  | 4.41 | 1.30, 14.9 | 0.017 |  |  | 1.26 | 0.99, 1.61 | 0.061 |  |  | 0.90 | 0.35, 2.32 | 0.829 |  |  |
| Family Hx of osteoporosis |  |  |  | 0.973 | 0.0000 |  |  |  | 0.761 | 0.0022 |  |  |  | 0.428 | 0.0019 |  |  |  | 0.252 | 0.0159 |
| No | 1.00 | — |  |  |  | 1.00 | — |  |  |  | 1.00 | — |  |  |  | 1.00 | — |  |  |  |
| Yes | 1.02 | 0.25, 4.18 | 0.973 |  |  | 0.74 | 0.10, 5.53 | 0.772 |  |  | 1.29 | 0.70, 2.37 | 0.411 |  |  | 1.94 | 0.68, 5.51 | 0.213 |  |  |
| Education |  |  |  | 0.733 | 0.0045 |  |  |  | 0.482 | 0.0338 |  |  |  | 0.991 | 0.0001 |  |  |  | 0.391 | 0.0315 |
| None/pre-secondary | 1.00 | — |  |  |  | 1.00 | — |  |  |  | 1.00 | — |  |  |  | 1.00 | — |  |  |  |
| Secondary/further education | 1.13 | 0.75, 1.70 | 0.569 |  |  | 0.56 | 0.19, 1.67 | 0.299 |  |  | 1.00 | 0.77, 1.30 | 0.998 |  |  | 0.51 | 0.15, 1.67 | 0.263 |  |  |
| Higher education | 0.90 | 0.44, 1.82 | 0.761 |  |  | 1.16 | 0.37, 3.63 | 0.802 |  |  | 0.97 | 0.57, 1.63 | 0.894 |  |  | 0.59 | 0.14, 2.55 | 0.481 |  |  |
| Dietary Ca meeting RNI |  |  |  | 0.089 | 0.0008 |  |  |  | 0.464 | 0.0115 |  |  |  | 0.221 | 0.0047 |  |  |  | 0.776 | 0.0012 |
| No | 1.00 | — |  |  |  | 1.00 | — |  |  |  | 1.00 | — |  |  |  | 1.00 | — |  |  |  |
| Yes | 0.62 | 0.37, 1.05 | 0.073 |  |  | 1.37 | 0.59, 3.16 | 0.460 |  |  | 1.26 | 0.86, 1.83 | 0.234 |  |  | 1.10 | 0.56, 2.17 | 0.774 |  |  |
| Dietary vitamin D intake (ug/1000 kcal) | 1.07 | 0.84, 1.35 | 0.576 | 0.580 | 0.0022 | 1.55 | 0.62, 3.89 | 0.352 | 0.414 | 0.0084 | 1.06 | 0.92, 1.22 | 0.418 | 0.422 | 0.0019 | 1.04 | 0.28, 3.90 | 0.956 | 0.956 | 0.0000 |
| Vegetable consumption (g/100 kcal/d) | 1.01 | 0.98, 1.04 | 0.577 | 0.582 | 0.0024 | 1.01 | 0.97, 1.06 | 0.606 | 0.625 | 0.0040 | 1.00 | 0.98, 1.02 | 0.898 | 0.898 | 0.0001 | 0.99 | 0.95, 1.03 | 0.633 | 0.616 | 0.0039 |
| Fruit consumption (g/100 kcal/d) | 1.00 | 0.98, 1.03 | 0.931 | 0.932 | 0.0001 | 1.01 | 0.97, 1.05 | 0.715 | 0.726 | 0.0021 | 1.00 | 0.99, 1.02 | 0.707 | 0.709 | 0.0005 | 0.98 | 0.94, 1.02 | 0.240 | 0.221 | 0.0246 |
| Ethanol consumption (units/d) |  |  |  | 0.518 | 0.0097 |  |  |  | 0.590 | 0.0062 |  |  |  | 0.100 | 0.0152 |  |  |  |  |  |
| None | 1.00 | — |  |  |  | 1.00 | — |  |  |  | 1.00 | — |  |  |  |  |  |  |  |  |
| >0 to <2 UK; >0 HK | 0.78 | 0.47, 1.29 | 0.325 |  |  | 0.76 | 0.28, 2.07 | 0.599 |  |  | 0.80 | 0.62, 1.04 | 0.100 |  |  |  |  |  |  |  |
| ≥2 UK | 0.95 | 0.52, 1.74 | 0.870 |  |  |  |  |  |  |  | 0.59 | 0.33, 1.05 | 0.074 |  |  |  |  |  |  |  |
| Use of Ca supplement |  |  |  | 0.555 | 0.0027 |  |  |  | 0.722 | 0.0027 |  |  |  | 0.833 | 0.0001 |  |  |  | 0.329 | 0.0126 |
| No | 1.00 | — |  |  |  | 1.00 | — |  |  |  | 1.00 | — |  |  |  | 1.00 | — |  |  |  |
| Yes | 0.58 | 0.08, 4.20 | 0.590 |  |  | 0.77 | 0.18, 3.34 | 0.731 |  |  | 0.93 | 0.49, 1.77 | 0.835 |  |  | 1.42 | 0.72, 2.83 | 0.314 |  |  |
| HRT use |  |  |  |  |  |  |  |  |  |  |  |  |  | 0.293 | 0.0035 |  |  |  | 0.463 | 0.0084 |
| Never |  |  |  |  |  |  |  |  |  |  | 1.00 | — |  |  |  | 1.00 | — |  |  |  |
| Past/current |  |  |  |  |  |  |  |  |  |  | 1.22 | 0.83, 1.79 | 0.304 |  |  | 0.55 | 0.13, 2.39 | 0.427 |  |  |
| *^1^* HR = Hazard Ratio, CI = Confidence Interval | | | |  |  |  | | |  |  |  | | |  |  |  | | |  |  |
