# Supplementary figures and images for: Fracture Incidence and the Relevance of Dietary and Lifestyle Factors Differ in the United Kingdom and Hong Kong: An International Comparison of Longitudinal Cohort Study Data
Source: Calcif Tissue Int. 2021 Jun 3;109(5):563–76. doi: 10.1007/s00223-021-00870-z (PMC8484188; doi:10.1007/s00223-021-00870-z)

## Slide 1
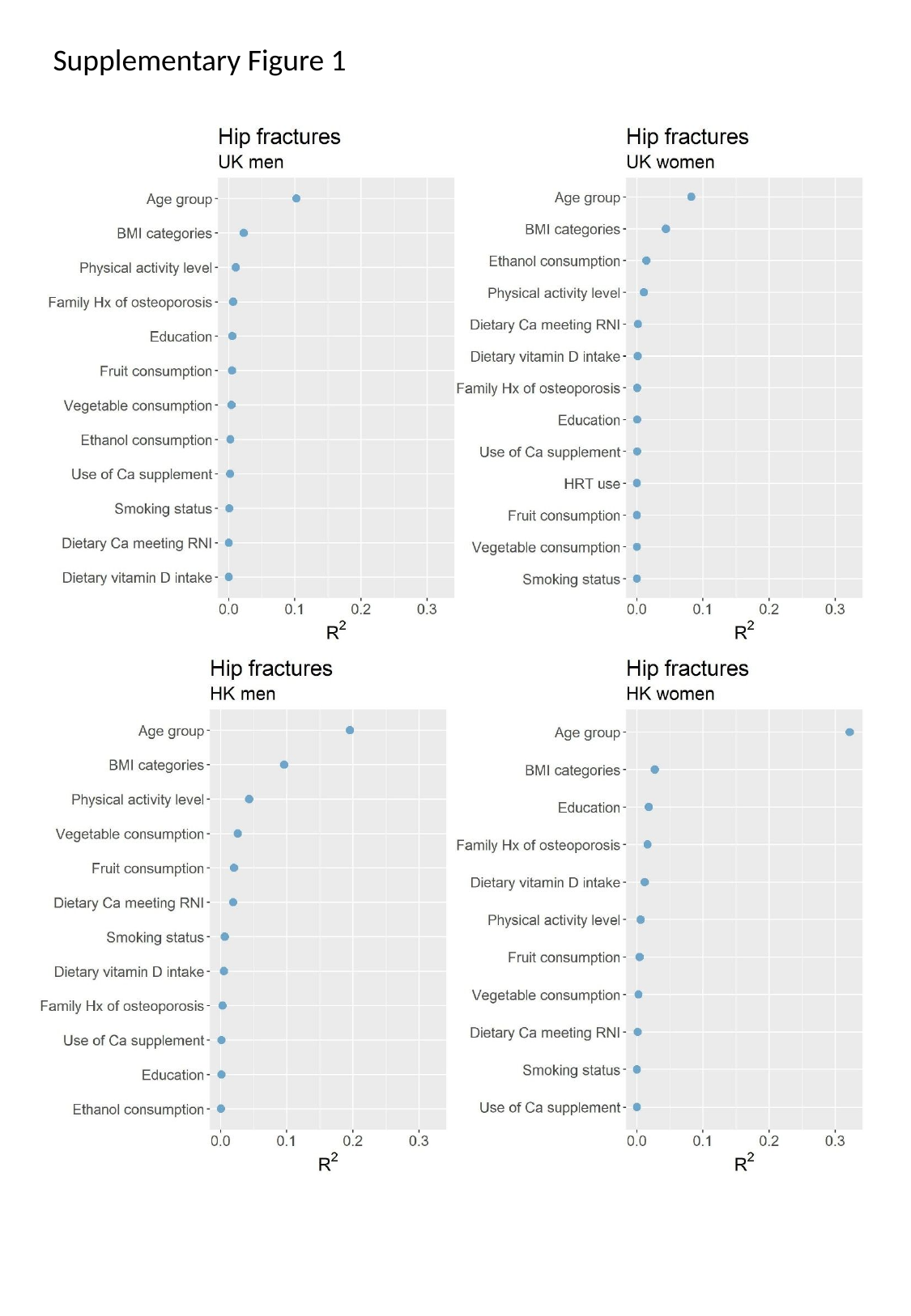

Supplementary Figure 1

Supplement: Supplementary file 6 — Supplementary file6 (PPTX 451 kb) [file 223_2021_870_MOESM6_ESM.pptx]

## Slide 1
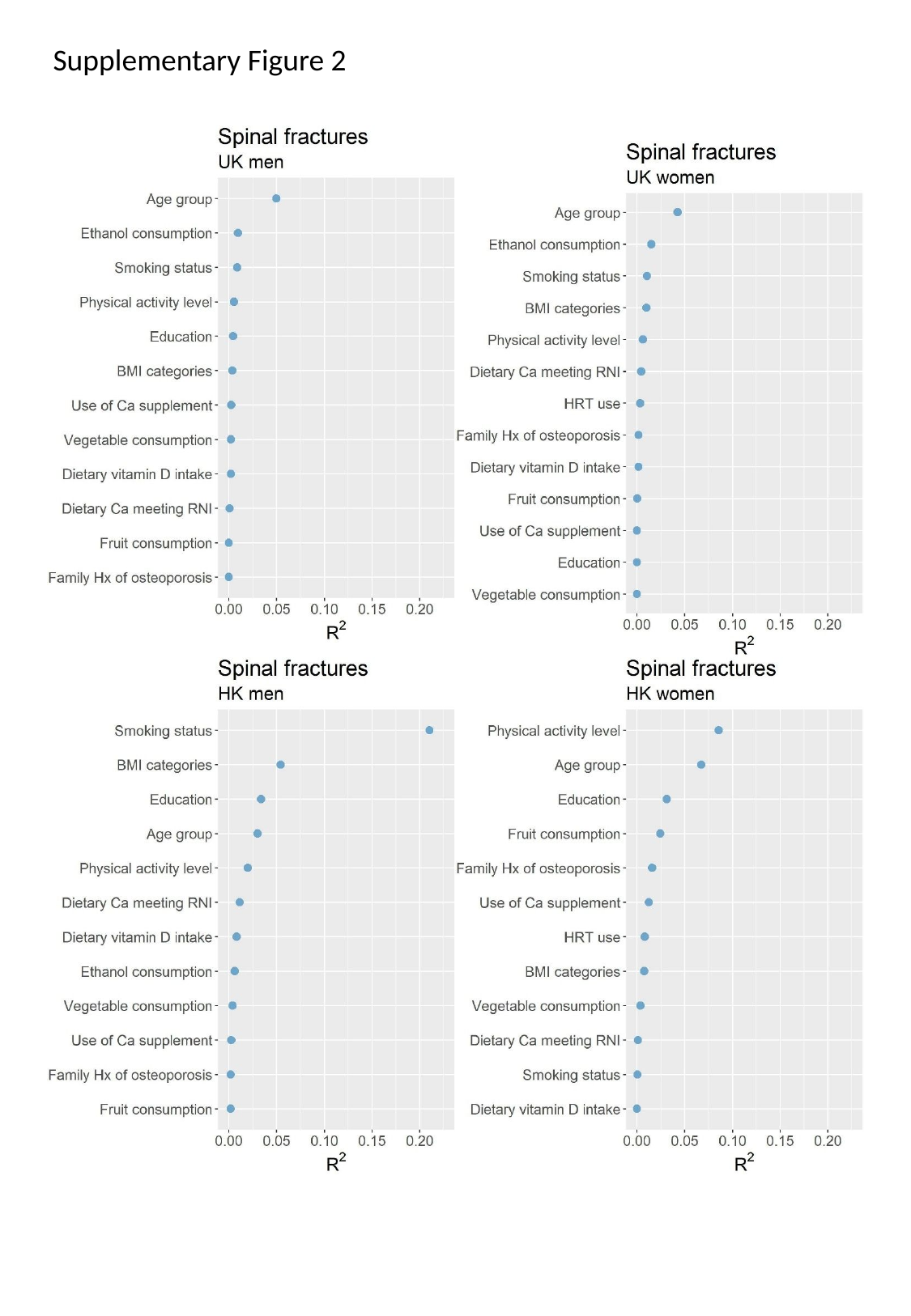

Supplementary Figure 2

Supplement: Supplementary file 7 — Supplementary file7 (PPTX 481 kb) [file 223_2021_870_MOESM7_ESM.pptx]

## Slide 1
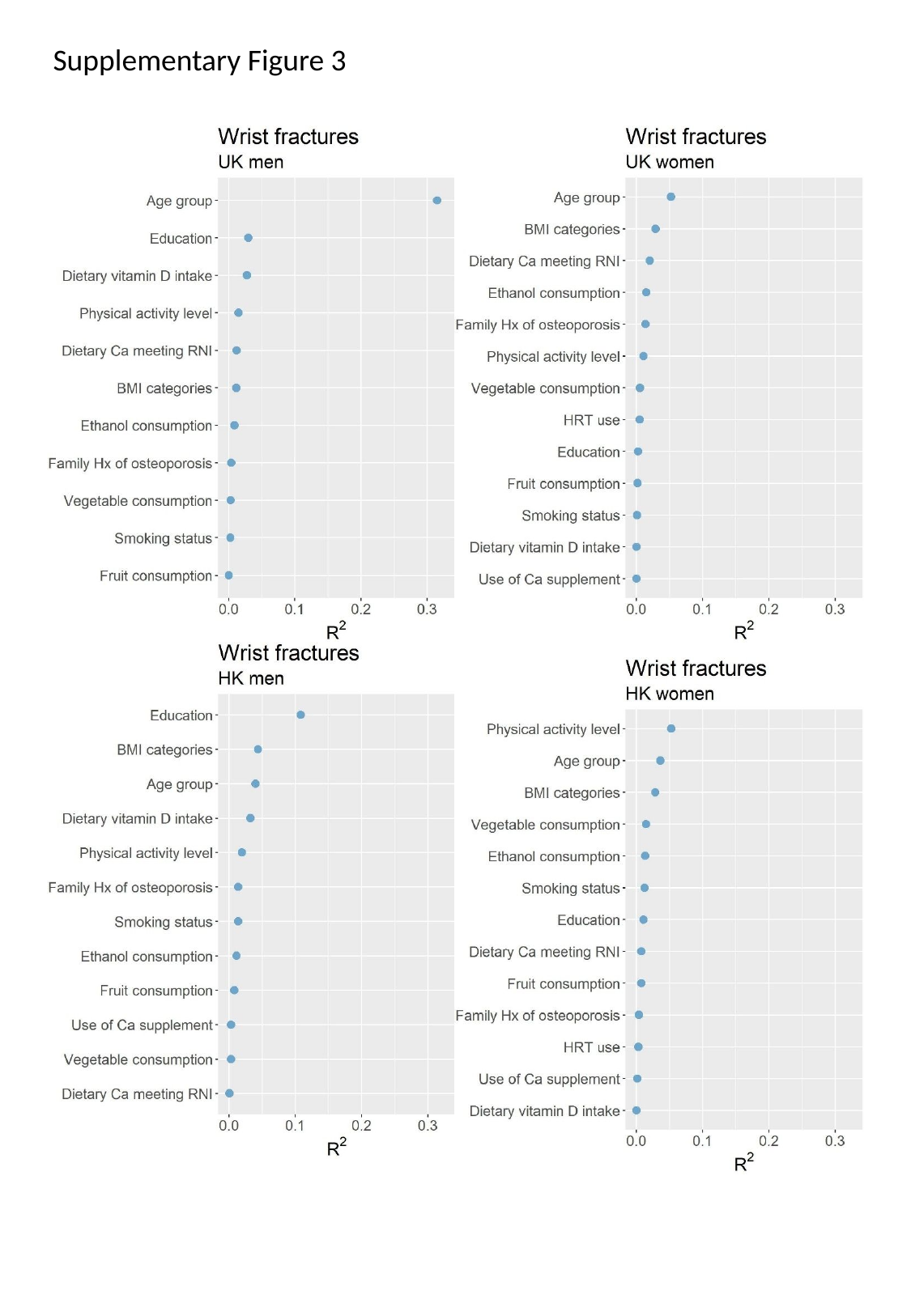

Supplementary Figure 3

Supplement: Supplementary file 8 — Supplementary file8 (PPTX 458 kb) [file 223_2021_870_MOESM8_ESM.pptx]
